# A banana transcriptional repressor MaAP2a participates in fruit starch degradation during postharvest ripening

**DOI:** 10.3389/fpls.2022.1036719

**Published:** 2022-11-11

**Authors:** Yunyi Xiao, Ying Li, Lejun Ouyang, Aiguo Yin, Bo Xu, Ling Zhang, Jianye Chen, Jinfeng Liu

**Affiliations:** ^1^ College of Biological and Food Engineering, Guangdong University of Petrochemical Technology, Maoming, China; ^2^ College of Horticultural Science, South China Agricultural University, Guangzhou, China

**Keywords:** banana fruit, postharvest ripening, APETALA2, starch degradation, transcriptional repressor

## Abstract

Fruit postharvest ripening is a crucial course for many fruits with significant conversion of biosubstance, which forms an intricate regulatory network. Ethylene facilitates the ripening process in banana with a remarkable change of fruit starch, but the mechanism adjusting the expression of starch degradation-related enzyme genes is incompletely discovered. Here, we describe a banana APETALA2 transcription factor (MaAP2a) identified as a transcriptional repressor with its powerful transcriptional inhibitory activity. The transcriptional level of MaAP2a gradually decreased with the transition of banana fruit ripening, suggesting a passive role of MaAP2a in banana fruit ripening. Moreover, MaAP2a is a classic nucleoprotein and encompasses transcriptional repressor domain (EAR, LxLxLx). More specifically, protein–DNA interaction assays found that MaAP2a repressed the expression of 15 starch degradation-related genes comprising *MaGWD1*, *MaPWD1*, *MaSEX4*, *MaLSF1*, *MaBAM1-MaBAM3*, *MaAMY2B*/*2C/3A/3C*, *MaMEX1*/*2*, and *MapGlcT2-1/2-2 via* binding to the GCC-box or AT-rich motif of their promoters. Overall, these results reveal an original MaAP2a-mediated negative regulatory network involved in banana postharvest starch breakdown, which advances our cognition on banana fruit ripening and offers additional reference values for banana varietal improvement.

## Introduction

For many fruits, postharvest ripening is a living and evolutive process that covers abundant metabolic changes contributing to fruit edibility. Remarkably, starch degradation plays a significant role in fruit postharvest ripening, especially in starch-rich fruits, such as banana and mango (Patino-Rodriguez et al., 2020; Zhu et al., 2021). Starch is an important storage carbohydrate of fruits, consisting of two types of glucose polymers (amylopectin 75%–90% and amylose 10%–25%) (Zhu et al., 2021). The former is a straight chain molecule without branches constituted by glucose residue polymerization with α-1,4-glycosidic bonds from the beginning to the end, while the latter is a long chain with branches composed of glucose residue polymerization with bonds of α-1,4-glycosidic and α-1,6-glycosidic. The interaction of amorphous amylose and amylopectin eventually forms semicrystalline granules that are insoluble in water, namely, starch granules (Seung and Smith, 2019).

Starch can be divided into temporary starch and storage starch according to its functions. Temporary starch is distributed in the “source” tissues such as leaves, which is formed by photosynthesis by day and degraded by night to provide substrates for leaf respiration and further sucrose synthesis and transferred to the “reservoir” of plants (Kötting et al., 2010). Storage starch is distributed in fruits, seeds, roots, tubers, bulbs, and other non-photosynthetic cell plasmids (amyloplast), which are used for medium- or long-term storage. When the time is right, storage starch will be redegradable and utilized. For example, the energy required for seed germination of cereals was mainly obtained by hydrolysis of stored starch (Zhang et al., 2021). The main direct products of starch degradation are glucose and maltose, which can be transported through the plastid membrane for cell metabolism and utilization. First, starch degradation maintains a continuous carbohydrate supply for non-photosynthetic organs, which is essential for the normal cycle of plant growth and adaptation to the growth environment (Tsamir-Rimon et al., 2021). Furthermore, starch degradation is crucial for fruit flavor formation, a transition from tasteless or sour to sweet, as well as fruit respiration, especially climacteric fruit, which provides a sufficient energy substrate for the climacteric during postharvest ripening (Zhu et al., 2021).

Studies on leaf starch degradation in *Arabidopsis thaliana* show that temporary starch degradation is accomplished by the co-participation of multiple enzymes (Zeeman et al., 2010). In this complex project, firstly, temporary starch is phosphorylated by combined actions of GWD (glucan, water dikinase) and PWD (phosphoglucan, water dikinase), which breaks the semicrystalline granule surface for starch degradation initiation (Kötting et al., 2005; Hejazi et al., 2010; Adegbaju et al., 2020). Then, glucans are dephosphorylated by phosphoglucan phosphatases starch excess 4 (SEX4) and like SEX four 1/2 (LSF1/2) (Silver et al., 2014; Wilkens et al., 2016). Significantly, the phosphorylation and dephosphorylation of starch let the whole hydrolyzation achievable by a string of hydrolytic enzymes, such as β-amylase (BAM) (Monroe and Storm, 2018; Han et al., 2021), isoamylase (ISA) (Sundberg et al., 2013), and 4-α-glucanotransferase disproportionating enzyme (DPE) (Malinova and Fettke, 2017; Smirnova et al., 2017). Last, membrane channel protein MEX (maltose transporter) and pGlcT (glucose transporter) transport the catabolites (glucose and maltose) to cytosol, providing sufficient substrates for respiration and secondary metabolism (Reidel et al., 2008; Cho et al., 2011). Basic research on starch degradation in *Arabidopsis* leaves provides sufficient reference value for other model plants and fruit storage starch degradation.

Starch degradation mechanism analysis has been incipiently studied in starchy fruits during ripening. Soluble sugar was accumulated for fruit sweetening through a carbon source provided by starch degradation with the combined actions of starch granule surface hydrolytic enzymes alpha- and beta-amylases during mango ripening (Peroni et al., 2008). Moreover, in kiwifruit, analysis of 24 potential genes that might conduce to starch degradation showed that *AdAGL3*, *AdAMY1*, and *AdBAM3.1/3L/9* played a prominent role in fruit starch degradation (Hu et al., 2016). In addition, later research identified a zinc finger transcription factor (TF), AdDof3, which bonds compactly with the *AdBAM3L* promoter to upregulate *AdBAM3L* expression in kiwifruit (Zhang et al., 2018). By the way, transgenic kiwifruit leaves with decreased starch content for stable overexpression of *AdBAM3L*, indicating that *AdBAM3L* was a crucial member of starch degradation and AdDof3 mediated starch degradation by transcription control of AdBAM3L (Zhang et al., 2018).

Banana is an economically important fruit crop with a classic postharvest climacteric ripening. It is also well-known for its rich starch that reaches 20%–25% by raw weight of the fruit pulp followed by decreases to 5% after postharvest ripening (Do Nascimento et al., 2006) . In the beginning, immunofluorescence microscopy verified that α-amylase and β-amylase participated in the process of starch granule degradation in banana ripening (Peroni-Okita et al., 2013). Previous studies had shown 27 candidates for banana postharvest starch degradation (Xiao et al., 2018). Furthermore, 18 starch granule surface-bound enzymes associated with starch degradation were identified *via* iTRAQ-based proteomics method, of which 10 enzymes were meaningfully upregulated during postharvest ripening (Xiao et al., 2018). Later, the research identified two TFs, MabHLH6 and MaMYB3, that acted respectively as a positive and negative director of starch degradation by explicitly binding the promoters of key starch degradation genes in banana fruit (Fan et al., 2018).

In banana, an integrated comprehension of regulatory networks manipulating starch degradation is not yet sufficient, especially for the vital roles of TFs in gene transcriptional control. In the promoters of key functional genes associating with fruit ripening, there are abundant specific DNA sequence motifs for interaction with corresponding TFs, which provide the essential regulation chances for TFs during the developmental processes. A recent study successively found that MaEIL2, MaMYB16L, MaARF2/12/24, MaMYB3/308, and MaMADS36/55 took part in the regulation of banana fruit starch degradation (Song et al., 2019; Miao et al., 2020; Jiang et al., 2021; Liu et al., 2021; Zhu et al., 2021). Thus, it can be seen that the starch degradation process in starchy banana fruits during ripening is complex and regulated multidimensionally. In this study, sequence analysis showed that AT-rich and GCC motifs were rich in the key promoters of starch degradation-related genes. Furthermore, we identified an APETALA2 (AP2) TF, MaAP2a, directly targeting the promoters of *MaGWD1*, *MaPWD1*, *MaSEX4*, *MaLSF1*, *MaBAM1/2/3*, *MaAMY2B*/*2C*/*3A*/*3C*, *MaMEX1*, *MaMEX2*, and *MapGlcT2-1*/*2-2 via* the GCC-box or AT-rich DNA motifs. Our work confirms an AP2 TF suppressing starch degradation-related gene expression, thus negatively impacting fruit starch degradation. These findings facilitate the comprehension of intricate starch degradation regulatory networks, a crucial process of fruit ripening and fruit commodity value formation in banana.

## Materials and methods

### Materials

Banana materials were employed, the same set of previous experimental materials published on *Plant Biotechnology Journal*, displaying three postharvest banana ripening processes (natural ripening, ethylene-induced ripening, 1-MCP-delayed ripening, and the ethylene production of fruits reached a maximum at days 18, 3, and 30, respectively) (Xiao et al., 2018). Tobacco (*Nicotiana benthamiana*) was cultivated in a growth illumination incubator of 23°C under duration-controlled conditions (18-h light/6-h dark), until plants with 8–12 healthy leaves were singled out for transient expression assays mediated by *Agrobacterium tumefaciens*.

### Total RNA extraction and analysis of gene expression

Hot borate method was selected to isolate high-quality total RNA from the least three parallel fruit pulps (Wan and Wilkins, 1994). Integrality of isolated RNA was measured by 1.2% agarose gel electrophoresis with that resulted in two electrophoretic bands (28S/18S) without degradation under UV (**Supplementary Figure S3**). Quantification of RNA was performed using ultramicro spectrophotometer (ATPIO, Nanjing, China) with three duplicates. The extracted 2 µg total RNA was utilized for cDNA synthesis using Hifair^®^ III 1st Strand cDNA Synthesis SuperMix for qPCR (gDNA digester plus) (Yeasen, Shanghai, China). Quantitative real-time PCR (qRT-PCR) was performed using CFX96 Touch Real-Time PCR Detection System (Bio-Rad, CA, USA) using the Hieff UNICON^®^ Power qPCR SYBR Green Master Mix (antibody technique, No Rox) (Yeasen, Shanghai, China) following the product manual. The housekeeping gene *MaRPS2* was used to normalize the expression values in terms of a previous report (Chen et al., 2011). Gene primers for qRT-PCR are shown in **Supplementary Table S1**.

### Subcellular localization

The full-length Coding sequence (CDS) regions of MaAP2a were cloned into pBE-GFP vector with a green fluorescent protein (GFP) tag in-frame on C-terminal, generating 35S::MaAP2a-GFP co-expression plasmids. Primers are listed in **Supplementary Table S1**. The recombinant plasmid and contrast (pBE-GFP) were transiently assimilated in 4-week-old tobacco (*N. benthamiana*) healthy leaves by *Agrobacterium* injection according to the method of Zhu et al. (2021), and GFP fluorescence was imaged by Zeiss Axioskop 2 Plus fluorescence microscope at 2.5 days after injection.

### Dual-luciferase reporter assay

There were 26 enzyme members involved in starch degradation of ripening fruit (Xiao et al., 2018). Reporters were constructed respectively by promoters of the 26 genes *via* a vector (pGreenII 0800-LUC), while MaAP2a was expressed by the vector (pEAQ) as an effector. Primers of plasmid constructs are listed in **Supplementary Table S1**. Effector paired one reporter were both transiently expressed in healthy leaves of *N. benthamiana* as previously reported (Hellens et al., 2005). The kit (Yeasen, Shanghai, China) for dual-luciferase assay was employed to assay the transient expression level of the reporter gene in injected leaves after 48 h. Filter Max F5 (Molecular Devices, CA, USA) was used for chemiluminescence determination of REN (*Renilla luciferase*) and LUC (firefly luciferase) as guideline. The transcriptional regulatory behavior of MaAP2a to targeted promoters is expounded by the LUC/REN ratio. At least five repeats of biology were performed for each group.

### Transcriptional activation activity *in vivo*

In order to analyze the transcriptional activity of MaAP2a, its full-length CDS was constructed into a vector with 35S promoter and a co-fusion expression GAL4BD (GAL4 DNA-binding domain) tag resulting in an effector (BD-MaAP2a). The artificially modified reporter vector carries LUC driven by 5× GAL4-binding motifs with minimal component of CaMV 35S promoter and includes a REN launched by CaMV 35S promoter as the internal control. Primers are shown in **Supplementary Table S1**. The effector and reporter were co-infiltrated into healthy leaves of tobacco as introduced above. The LUC/REN ratio represented the transcriptional ability of MaAP2a. The operation was implemented as described above.

### Protein purification and electrophoretic mobility shift assay

The coding region R2 (247-323 aa) of MaAP2a was cloned in-frame into pGEX-4T-1 vector [glutathione S-transferases (GST) tag]. Then, the GST-MaAP2a-R2 recombinant construct was assimilated into *Escherichia coli* Rosetta (DE3) strain. Protein-induced expression was conducted in a 200-ml medium with 1.0 mM isopropyl β-D-1-thiogalactopyranoside (IPTG) for 6 h *via* a shaker at 28°C. The fused tag protein was obtained using GSTPur Glutathione Kit (Smart-Lifesciences, Changzhou, China) following the manufacturer’s instructions.

The single-stranded ~59-bp fragments containing GCC-box (GCCGCC) or AT-rich (TTTGTT) starch degradation-related gene promoters were artificially synthesized and tagged by Biotin 3′ End DNA Labeling Kit (Beyotime, Shanghai, China) followed by double-stranded renaturation. In addition, the unlabeled double-stranded DNA fragment served as a competitor, while a mutation fragment changed within the mGCC-box (AAAAAA) and mAT-rich (TGTGTG) as a mutant contender in the analysis. Chemiluminescent EMSA Kit (Beyotime, Shanghai, China) was applied to EMSA in accordance with the manual. Chemiluminescence signal of sample was imaged on a ChemiDoc™ MP Imaging System (Bio-Rad, CA, USA). The fragment sequences used in the EMSA test are listed in **Supplementary Table S1**.

### Specific polyclonal antibody of MaAP2a preparation and detection of MaAP2a protein by Western blotting

The pET28a(+) (Novagen) was used as the protein expression vector of MaAP2a with full-length CDS building the His-MaAP2a in-frame structure. The recombinant protein expression was induced by *E. coli* strain BL21 (DE3). Then, the purified induced protein was used for antigen antibody induction. Immune antibody was obtained in rabbit by Sino Biological Company (Beijing, China). The involved primers are listed in **Supplementary Table S1**.

Total protein was extracted and purified from banana pulp followed by electrophoresis separated in Sodium dodecyl sulfate-polyacrylamide gel electrophoresis (SDS-PAGE) with 20-µg protein each lane. Then, the separated protein was transferred onto the NC membrane (Merck, 0.45 µm). Western blotting experiment was performed using anti-MaAP2a antibody and with secondary antibody IPKine™ Horseradish peroxidase (HRP), goat anti-rabbit immunoglobulin G (IgG) Heavy chain specificity (HCS) (Abbkine, Wuhan, China). The reference control was selected as anti-GADPH. Detailed operation was referred to that described by Xiao et al. (2018).

### Yeast one-hybrid assay

The Matchmaker™ Gold Yeast One-Hybrid (Y1H) System (Takara, Dalian, China) was employed to analyze the protein–DNA interaction. The promoter fragment (-1,147~-1,981) of MaGWD1 was cloned into the pAbAi plasmid. Then, the recombinant plasmid was linearized by restriction enzyme (*Bst*BI) and introduced into the receptor (Y1H Gold) to form a bait-specific reporter strain (*MaGWD1-pro-AbA^r^* Y1H). Primers designed are listed in **Supplementary Table S1**. In turn, AD-MaAP2a was introduced into the bait-specific reporter strain with AD empty gsensor as the negative control. The interaction of prey–bait was confirmed according to the survival of the transformants on medium [SD/-Leu with 200 ng/ml aureobasidin A (AbA)]. When the prey protein (AD-MaAP2a) binds to the bait motif, the GAL4 AD will promote the expression of *AbA^r^* that endows the transformants to survive on medium containing the minimal inhibitory concentration of AbA antibiotic.

### Chromatin immunoprecipitation–qPCR analysis

*In vivo* protein–DNA interaction analysis was carried out by chromatin immunoprecipitation (ChIP) as described by Fan et al. (2018). Unripe banana pulp was cross-linked by 1% formaldehyde and from which chromatin was obtained. The chromatin was fractured to ~500 bp in length by sonication, while anti-MaAP2a antibody was used for specific immunoprecipitation. The immunoprecipitation DNA by anti-MaAP2a antibody was measured *via* RT-qPCR in triplicate. Internal negative control (IgG) was utilized to calculate the signal intensity of ChIP-qPCR. The primers of RT-qPCR are listed in **Supplementary Table S1**.

## Results

### MaAP2a was a novel MaGWD1 promoter-interacting protein

**Figure 1:**
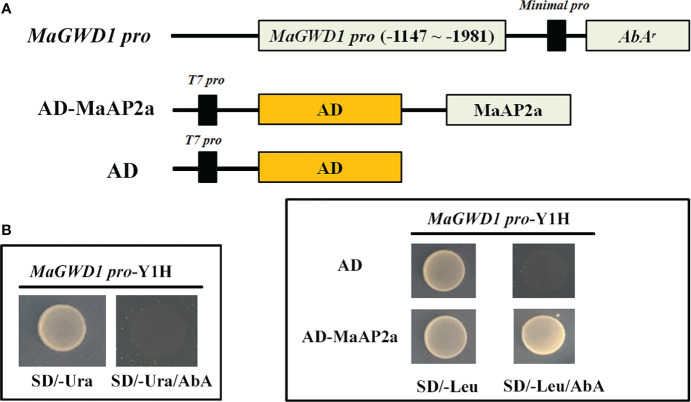
Binding of MaAP2a to *MaGWD1* promoter by yeast one-hybrid (Y1H) assay. **(A)** Schematic diagram of the plasmids used in the Y1H assay. **(B)** Y1H analysis of MaAP2a binding to *MaGWD1* promoters. Left: No survival of the bait (*MaGWD1-*promoter Y1H) on synthetically defined (SD) medium lacking uracil (Ura) in the presence of minimal inhibitory concentration of aureobasidin A (AbA). Right: Yeast growth assay after the Y1H reporter strains were transformed with plasmids carrying cassettes constitutively expressing Activation domain (AD)-MaAP2a effector or empty AD (negative control). Interaction was confirmed based on the normal growth of the transformed yeast on SD medium lacking leucine (Leu) in the presence of AbA.

Previous work had proposed that MaGWD1 played a vital role in starch degradation of postharvest banana fruit consistent with ethylene-induced fruit ripening (Xiao et al., 2018). To ulteriorly discover the potential regulators of *MaGWD1*, one-hybrid cDNA library screening was performed with a bait (*MaGWD1* promoter) and a prey (banana ripening-related cDNA library). Through strict screening, an AP2 TF, named MaAP2a (XP_009418515.1), was identified to target the promoter of *MaGWD1*. MaAP2a bound to the *MaGWD1* promoter was verified by Y1H assay once again (**Figure 1**).

### Molecular characterization of MaAP2a

**Figure 2:**
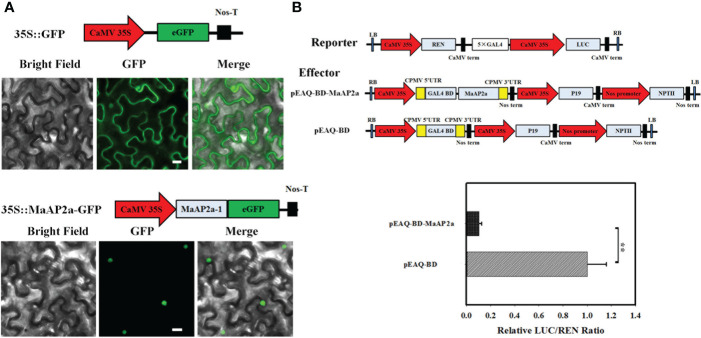
Subcellular localization and transcriptional inhibitory activity of MaAP2a in tobacco leaves. **(A)** Subcellular localization of MaAP2a in tobacco epidermal cells. Green fluorescence was imaged by fluorescence microscopy in 2 days after infiltration. Scale bar = 25 μm. **(B)** Transcriptional activity of MaAP2a *in planta*. Compared with the pEAQ-BD control, pEAQ-BD-MaAP2a significantly restrained the expression of the LUC reporter. The ratio of LUC to REN of the pEAQ-BD vector was used as a calibrator (value set as 1). Values represent means of six biological replicates. The ** represents significant differences in p values <0.01.

**Figure 3:**
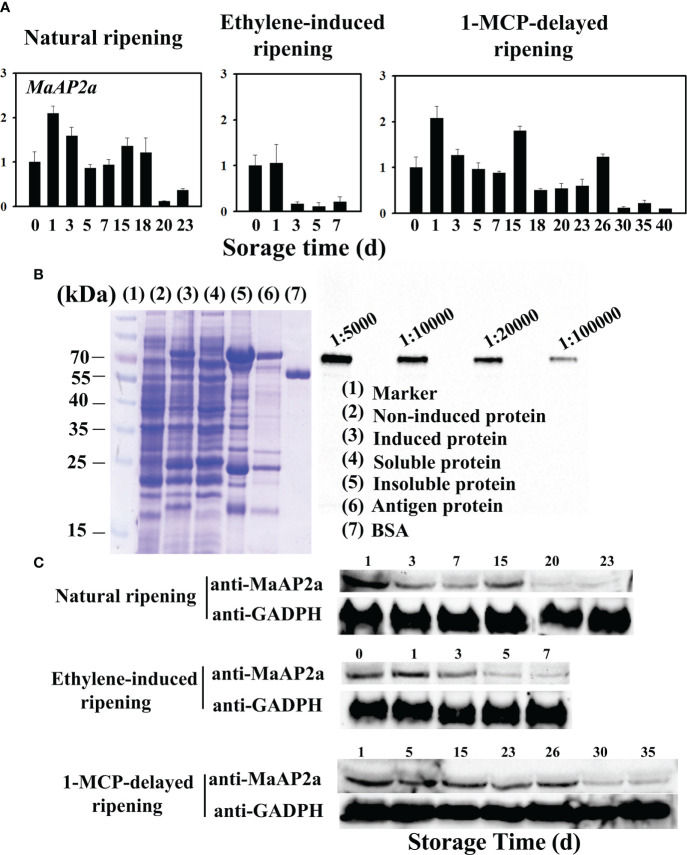
Gene transcriptional expression and translation protein profile of MaAP2a in banana fruit pulp in three different ripening behaviors. **(A)** The relative transcript levels of *MaAP2a* are shown as a ratio relative to the 0 day of natural ripening, which was set as 1. Each value represents the mean ± SE of three replicates. **(B)** Preparation of polyclonal antibody against MaAP2a and specificity analysis of antibody by Western blotting with four gradient concentrations of antibody from 1:5,000 to 1:100,000. **(C)** Western blot analysis of protein level of MaAP2a in banana pulp with three different ripening behaviors. Anti-GADPH antibody was used as a reference control to normalize the loading proteins.

The full-length cDNA of *MaAP2a* comprises an open reading frame of 1,278 bp in length, encoding a protein of 425 amino acids with 46.70 kDa calculated molecular weight and a predicted *p*I of 5.70. MaAP2a was clustered close to SlAP2a by alignment with all AP2 of banana (**Supplementary Figure S1**) and contained tow typical AP2 domain (**Supplementary Figure S2**). MaAP2a protein was localized in the nucleus (**Figure 2A**) as a classic TF and exhibited transcriptional inhibitory activity in tobacco leaf (**Figure 2B**). Contrary to *MaGWD1*, the transcription of MaAP2a showed a strong negative correlation with postharvest fruit ripening and starch degradation, with obvious decreases on days 20, 30, and 3 in natural, 1-MCP-delayed, and ethylene-induced ripening bananas, respectively (**Figure 3A**). Moreover, Western blotting with specific anti-MaAP2a antibody (**Figure 3B**) showed that MaAP2a protein likewise decreased at the ripening phase (**Figure 3C**).

### MaAP2a directly binds to the starch degradation-related gene promoters with suppression ability

**Figure 4:**
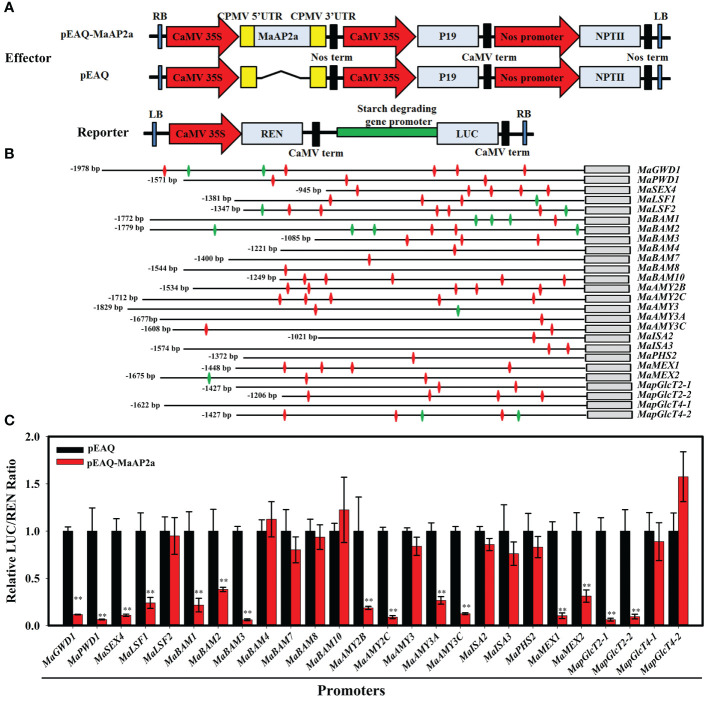
DLR™ Assays of the association of MaAP2a with starch degradation-related gene promoters. **(A)** Diagrams of the reporter and effector constructs used in the dual-luciferase reporter assay. **(B)** Schematics of the 26 promoters of genes involved in starch degradation and the annotated length of promoters. Promoter GCC-box and AT-rich motifs are indicated with green and red diamonds, respectively. **(C)** Transient assays on the inhibition of MaAP2a with the promoters in *Nicotiana benthamiana* leaves. The ratio of LUC to REN of the empty vector (pEAQ) plus promoter reporter was set as 1. Each value represents the means of six biological replicates, and vertical bars represent the SE. The ** represents significant differences in p values <0.01.

Based on a previous report, AP2 TF has two DNA-binding domains (AP2 domain) and the second AP2 domain binds a non-classical AT-rich motif as well as a GCC-box (Dinh et al., 2012). Besides *MaGWD1*, there were 25 other genes (*MaPWD1*, *MaSEX4*, *MaLSF1*, *MaLSF2*, *MaBAM1/2/3/4/7/8/10*, *MaAMY2B*/*2C*/*3*/*3A*/*3C*, *MaISA2/3*, *MaPHS2*, *MaMEX1*/*2*, *MapGlcT2-1/2-2*/*4-1*/*4-2*) positively related to banana starch degradation (Xiao et al., 2018). Hence, their promoters were chosen for identification of GCC-box (GCCGCC) and AT-rich (TTTGTT) motifs (**Figure 4B** and **Supplementary Material S1**). Then, the Dual-Luciferase^®^ Reporter (DLR™) assay system was performed to examine the potential capacity of MaAP2a to adjust these promoters. For DLR™ Assays, the vector offered an internal control of the REN reporter regulated by a promoter (CaMV 35S) and a LUC reporter driven by the promoters of starch degradation-associated genes and then was co-transformed with an overexpression vector carrying MabAP2a controlled by the CaMV 35S promoter in tobacco leaves, respectively (**Figure 4A**). Among the 26 DLR™ Assays shown in **Figure 4C**, activities of *MaGWD1*, *MaPWD1*, *MaSEX4*, *MaLSF1*, *MaBAM1/2/3*, *MaAMY2B*/*2C*/*3A*/*3C*, *MaMEX1*/*2*, and *MapGlcT2-1*/*2-2* promoters were observably repressed in the existence of MaAP2a, with a dramatic reduction in LUC/REN ratio in reference to the control.

**Figure 5:**
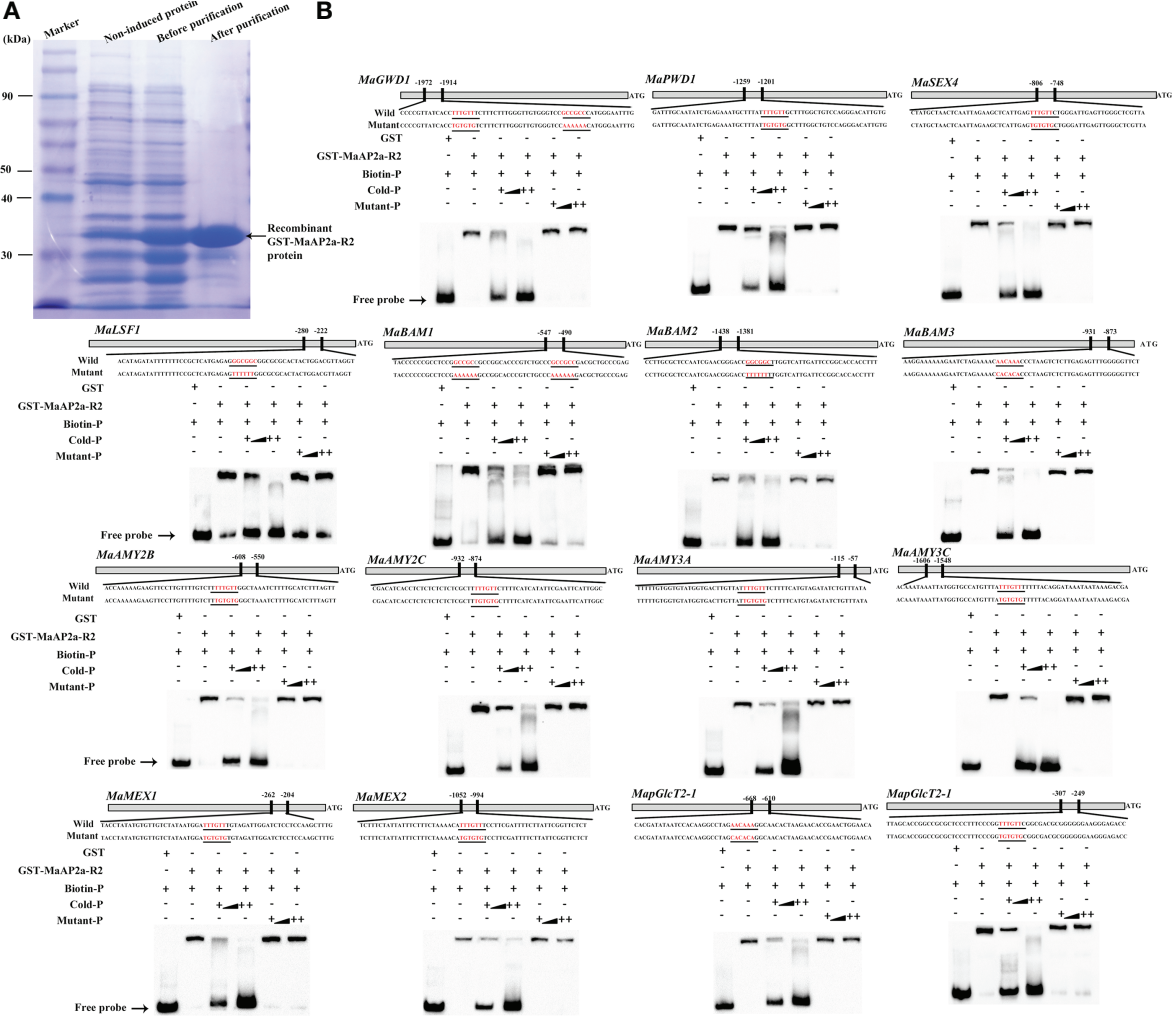
MaAP2a binds to the promoters of starch degradation-related genes *in vitro*. **(A)** Affinity purification of the recombinant GST-MaAP2a-R2 protein and SDS-PAGE gel dyed with Coomassie brilliant blue shows that the recombinant protein is suitable for the EMSA. **(B)** EMSA showing MaAP2a binding to the promoters of starch degradation-related genes containing GCC-box or AT-rich element. The probe sequences corresponding to each of the target gene promoters are shown, with red letters representing the GCC-box or AT-rich and their mutant. The purified GST or recombinant GST-MaAP2a-R2 protein was mixed with probes, and the protein–probe complexes were separated on native polyacrylamide gels. Triangles indicate increasing amounts of unlabeled or mutated probes for competition.

**Figure 6:**
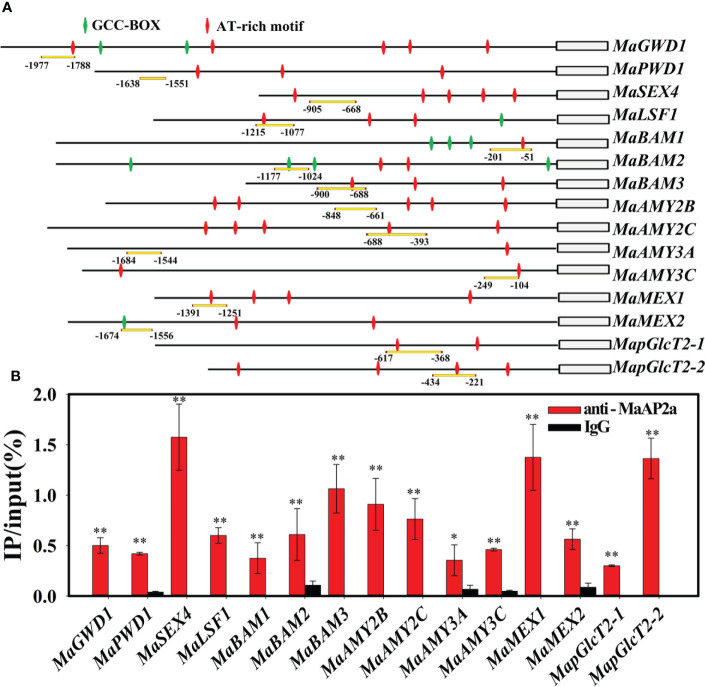
MaAP2a binds to the promoters of genes involved in starch degradation *in vivo*. **(A)** Schematic representation of selected regions for ChIP-qPCR analyses. Probes selected for the qPCR assay are indicated with yellow underline. GCC-box and AT-rich motifs of the promoters are indicated with green and red diamonds, respectively. **(B)** ChIP-qPCR assay showing the binding of MaAP2a to the promoters of starch degradation-associated genes. Nucleoprotein and DNA cross-linking complexes were extracted and immunoprecipated with MaAP2a antibody or IgG (negative control). The * and ** indicate significant differences in p values <0.05 or <0.01, respectively.

Furthermore, EMSA was employed to further verify the direct binding capacity to 15 gene promoters of MaAP2a. As illustrated in **Figure 5B**, the recombinant MaAP2a-R2 protein by affinity purification (**Figure 5A**) was capable of binding motifs of these promoter fragments which prompted shifts of electrophoretic mobility. Moreover, the shifted bands petered out by the increasing addition of unlabeled competitors (identical sequence with no label) but not by the addition of the mutated probes. Finally, *in vivo* binding of MaAP2a with these promoters was further confirmed by ChIP-qPCR method utilizing polyclonal anti-MaAP2a antibody. As expected, the promoter fragments with probes (**Figure 6A**) of 15 genes were notably enriched by the anti-MaAP2a contrasted with the negative control IgG (**Figure 6B**). Together, these data illustrated that MaAP2a might act as a negative transcriptional repressor of starch degradation-related genes by directly targeting their promoters.

## Discussion

Starch degradation of banana fruit is a meaningful and fateful biological process comprising multiple important enzymatic activities (**Supplemental Table S2**), such as GWD, PWD, BAM, and AMY, and increasing proofs reveal that ripening-related TFs build an intricate regulatory network to modulate changes of starch (Xiao et al., 2018; Song et al., 2019; Miao et al., 2020; Jiang et al., 2021; Liu et al., 2021; Zhu et al., 2021). Based on the well-defined role of GWD in the initiation of leaf temporary starch degradation, banana MaGWD1 that was consistent with fruit ripening also played a great job in starch degradation (Xiao et al., 2018). Our studies used Y1H system that *MaGWD1* promoter was arranged as a bait and screened for possible interacted TFs. One AP2 TF, MaAP2a, was originally identified followed by confirming its direct binding capacity to *MaGWD1* (**Figure 1**). In an effort to identify the obtained *MaAP2a* gene, further analysis affirmed its classic nucleoprotein of nuclear localization (**Figure 2A**) and with a remarkable transcriptional inhibitory activity (**Figure 2B**). Moreover, the expression of *MaAP2a* was found to be restrained observably during fruit ripening with ethylene (**Figure 3**), which was contrary to the expression of starch degradation-related genes (Xiao et al., 2018).

AP2 was originally recognized as a floral homeotic gene required for A-function with regard to the ABC model of floral development in *Arabidopsis* (Komaki et al., 1988) and belonged to a subfamily of AP2/ERF plant-specific TFs (Wollmann et al., 2010). Interestingly, AP2 TF has two DNA-binding domains, and its second one is responsible for DNA recognition sequence of AT-rich and GCC motif while the first one is lacking specific binding capabilities (Dinh et al., 2012). In this context, we found that MaAP2a bound downstream gene promoters by its second AP2 domain (AP2-R2) (**Figure 5B**), which was consistent with previous reports.

More and more reports confirm that AP2 TF plays a significant role in regulating a diverse range of plant developmental processes besides flowering, such as fruit development and substance metabolism (Chung et al., 2010; Ripoll et al., 2011; Patil et al., 2019; Jiang et al., 2020; Ding et al., 2021; Ding et al., 2022). In tomato, a climacteric fruit based on the noticeable induction of ethylene and respiration at the beginning of ripening, there was 5-fold enhancive ethylene in *SlAP2a* RNAi fruit during ripening, suggesting that SlAP2a is a negative regulatory TF to ethylene biosynthesis in maturing fruit. SlAP2a also altered carotenoid accumulation profiles by modifying the carotenoid pathway flux (Chung et al., 2010). In apple, MdSHN3 (AP2 TF) was demonstrated to be an activator of apple fruit cuticle formation and a suppressor of russet development (Lashbrooke et al., 2015). More recently, MdAP2-1a played a positive role in regulating anthocyanin biosynthesis *via* binding directly to the promoter and protein sequences of MdMYB10, a key TF of apple anthocyanin biosynthesis (Ding et al., 2022). Furthermore, increasing evidence reveals that AP2 also plays a role in deciding seed mass (Jiang et al., 2019; Jiang et al., 2020; Luo et al., 2021). In the present work, our results reveal the possible role of MaAP2a in banana postharvest starch degradation by inhibiting the expression genes related to starch degradation. Here, in an effort to identify the function of MaAP2a for fruit starch degradation, further analysis of protein–DNA interaction was carried out. Thus, EMSA and ChIP-qPCR results clearly demonstrate that besides *MaGWD1*, MaAP2a could also bind the promoters of *MaPWD1*, *MaSEX4*, *MaLSF1*, *MaBAM1/2/3*, *MaAMY2B/2C*/*3A*/*3C*, *MaMEX1*/*2*, and *MapGlcT2-1*/*2-2 via* the GCC-box or AT-rich motif of those promoters (**Figures 5B**, **6B**). Additionally, transient expression experiments demonstrated that MaAP2a plays a repressive activity in its downstream genes (**Figure 4C**). Collectively, these observations afford supportive evidence that MaAP2a could directly repress fruit starch degradation by negatively controlling the expression of genes connected with starch degradation.

## Conclusions

In general, we confirmed a transcriptional repressor MaAP2a that participated in postharvest banana ripening with a negative influence on starch degradation accomplished by directly damping starch degradation-related genes. The experimental results here enhanced our comprehension of postharvest fruit starch degradation through a complex transcriptional regulatory network corresponding to fruit ripening.

## Data availability statement

The original contributions presented in the study are included in the article/**Supplementary Material**. Further inquiries can be directed to the corresponding author.

## Author contributions

JL and JC designed and guided the experiments; YX performed most of the experiments; YL carried out partial experiments; YX and YL finished the manuscript, and LO, AY, BX, and LZ gave suggestions and changes to the manuscript. All authors contributed to the article and approved the submitted version.

## Funding

This research was funded by the National Natural Science Foundation of China (31901733), and Guangdong University (Natural Science) Young Innovative Talents Project (2017KQNCX134), and Talent Introduction Project of Guangdong University of Petrochemical Technology (2018rc34).

## Acknowledgments

We thank Dr. George P. Lomonossoff (Department of Biological Chemistry, John Innes Centre, Norwich Research Park) for the generous gift of pEAQ vectors.

## Conflict of interest

The authors declare that the research was conducted in the absence of any commercial or financial relationships that could be construed as a potential conflict of interest.

## Publisher’s note

All claims expressed in this article are solely those of the authors and do not necessarily represent those of their affiliated organizations, or those of the publisher, the editors and the reviewers. Any product that may be evaluated in this article, or claim that may be made by its manufacturer, is not guaranteed or endorsed by the publisher.
