# Supplementary material for: A banana transcriptional repressor MaAP2a participates in fruit starch degradation during postharvest ripening
Source: Front Plant Sci. 2022 Nov 11;13:1036719. doi: 10.3389/fpls.2022.1036719 (PMC9691770; doi:10.3389/fpls.2022.1036719)
Supplement: Supplementary file 1 [file DataSheet_1.doc]

**Supplemental Figure S1** Phylogenetic tree of MaAP2a.

**Supplemental Figure S2** Sequence logo of the AP2 and EAR domain in MaAP2a.

**Supplemental Figure S3** Agarose gel electrophoresis of fruit isolated RNA

**Supplemental Table S1** Primers used in this study.

**Supplemental Table S2** Genes locus and names for publication.

**Supplemental** **Text S1** Nucleotide sequences of the promoters of starch degradation enzyme genes.


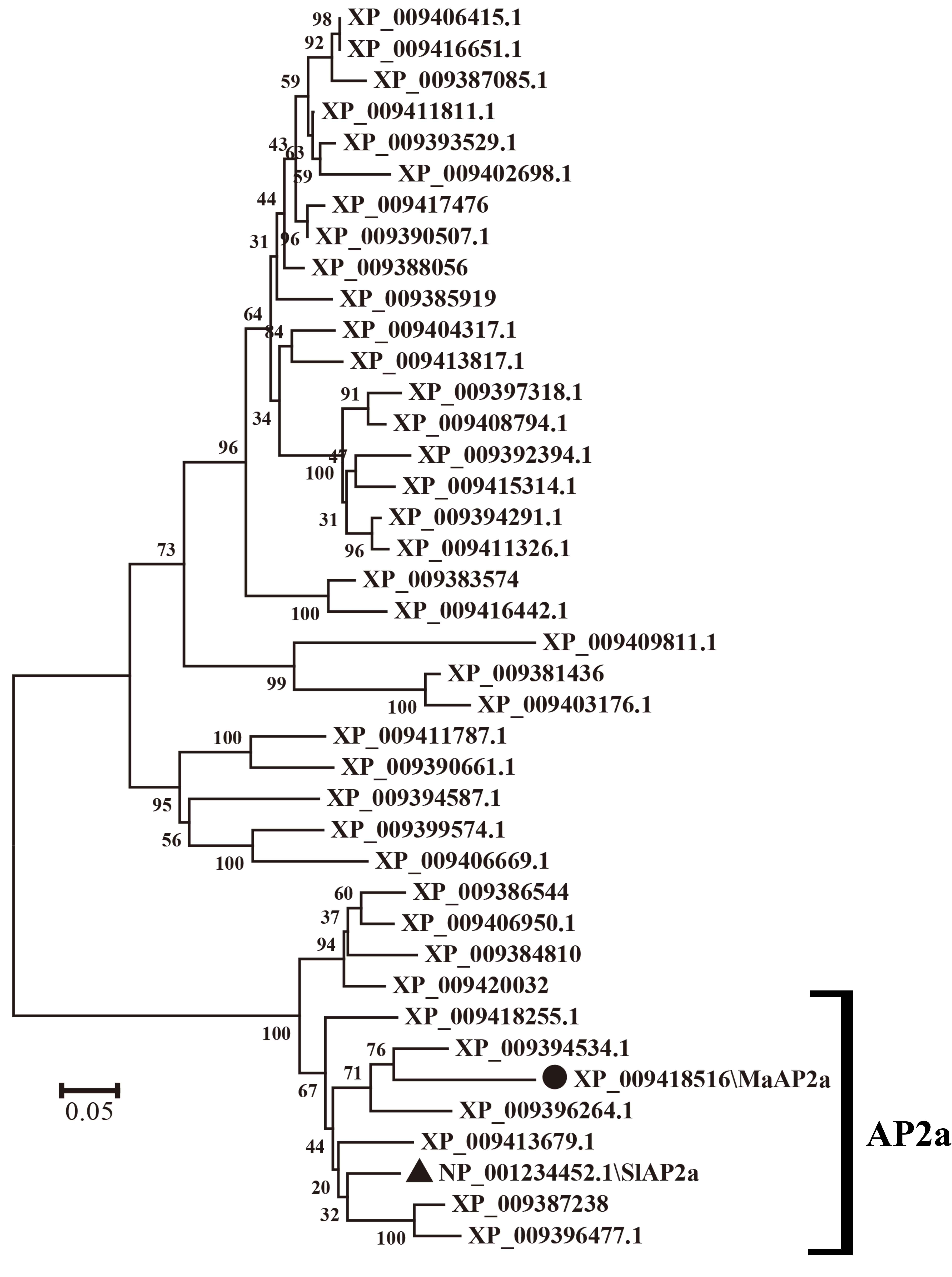


**Supplemental Figure S1** Phylogenetic tree of MaAP2. Banana MaAP2a (black circles) were aligned with other 38 AP2 members of the banana genome and tomato SlAP2a (black triangle). Multiple alignment was carried using CLUSTALW and the phylogenetic tree was constructed with MEGA6.0 using a bootstrap test of phylogeny with Neighbor-Joining test and default parameters.


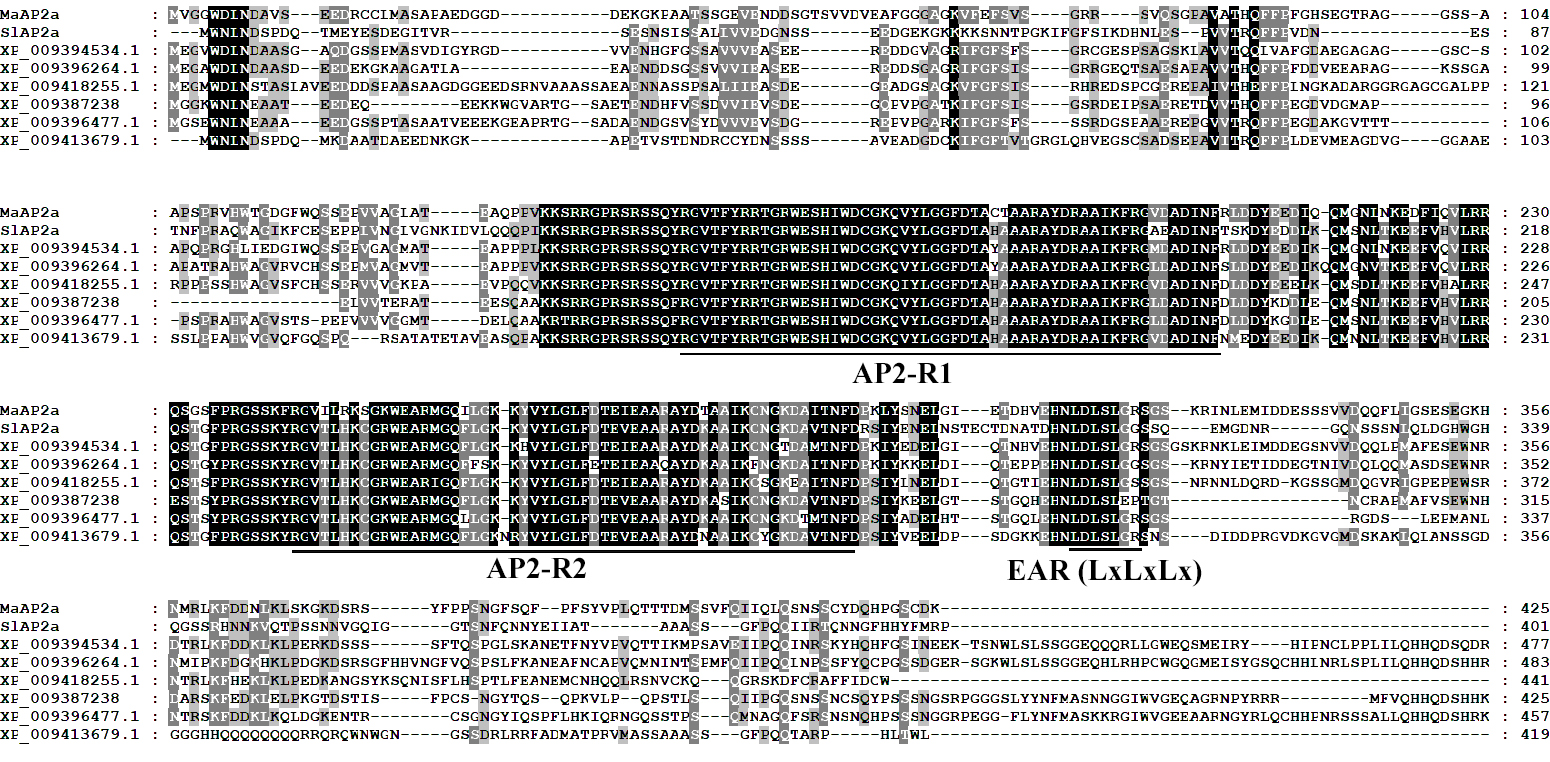


**Supplemental Figure S2** Sequence logo of the AP2 and EAR domain in MaAP2a. MaAP2a were aligned with the subfamily of AP2a proteins. The AP2-R2 amino acids in the basic domain that are important for DNA binding.


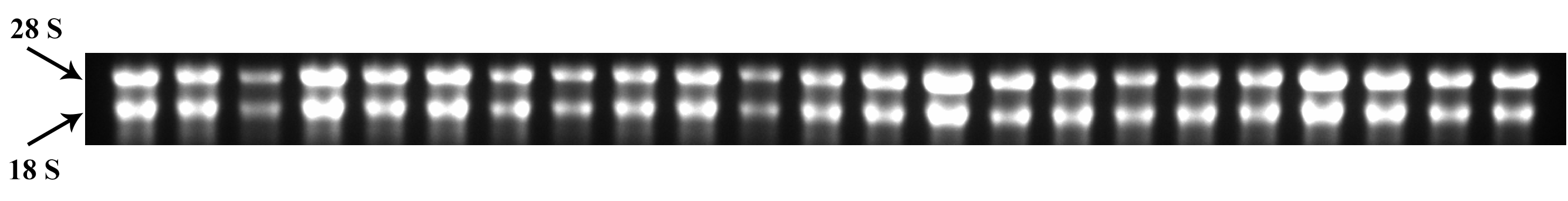


**Supplemental Figure S3** Agarose gel electrophoresis of fruit isolated RNA

**Supplemental Table S1** Summary of primers used in this study

| **Assay** | **Primer sequence (5’-3’)** | | | **Restriction Site** |
| --- | --- | --- | --- | --- |
| **Full length cloning** | ***MaAP2a-F: ATGGTCGGCGGGTGGGATCT***  ***MaAP2a-R: TTATTTATCGCAGCTGCCAG*** | | |  |
| **Subcellular localization** | ***MaAP2a-GFP-F:* gac*tctagagcagtcgacgATGGTCGGCGGGTGGGATCT***  ***MaAP2a-GFP-R:* cgggatccaccggtcgcc*TTTATCGCAGCTGCCAGGAT*** | | | ***Sal* I**  ***Bam*H I** |
| **RT-qPCR** | ***MaAP2a-qF: TAAGTGTAATGGGAAAGACGC*** | ***MaAP2a-qR: CTCGGAGCCTATGAGAAAT*** | |  |
| **Promoter isolation** | ***MaGWD1-pro-F: TCCACCGCCACCCCGTTATC***  ***MaPWD1-pro-F: CTACTGGCTCTTTGGGTAAAGT***  ***MaSEX4-pro-F: GATCAGATGTTATGGGAGGTC***  ***MaLSF1-pro-F: GCGTGGGGTCTAATGCAGTC***  ***MaLSF2-pro-F: CAGAACCCTGTGCTGATGGC***  ***MaBAM1-pro-F: TGCCCACCACCCTTACGAG***  ***MaBAM2-pro-F: CGGTAGCGACCTGGTTTGG***  ***MaBAM3-pro-F: GCTGTTGTTCAGTCTCTCATAG***  ***MaBAM4-pro-F: TCAGCCTCTGAGTTCTCCAC***  ***MaBAM7-pro-F: CAAGATTTATGGGAAGTGACG***  ***MaBAM8-pro-F: CTTGTTACACGACCACCAGC***  ***MaBAM10-pro-F: GGGTGATCTGACTTTGGAAC***  ***MaAMY2B-pro-F: CGTTTCCATGAGTGCCCGGTG***  ***MaAMY2C-pro-F: CATCTAAACGATCCATCGCG***  ***MaAMY3-pro-F: TGGGTTTGCTTCCGCTGGCC***  ***MaAMY3A-pro-F: GAATTGGCATCTATCAGTGCC***  ***MaAMY3C-pro-F: CGTTGACTGAGTTGTGCTACC***  ***MaISA2-pro-F: CAAGCATGGAACCAAAGAACT***  ***MaISA3-pro-F: GAGGCATGGATCCGAGGCTT***  ***MaPHS2-pro-F: CGTCATCAAGTTGGCCTTGT***  ***MaMEX1-pro-F: AGAGGTCAAGGTGGTGCT***  ***MaMEX2-pro-F: GCGTGGAGTTGATATGGGTG***  ***MapGlcT2-1-pro-F:CAAGATATCCTTAGTACCATGCAG***  ***MapGlcT2-2-pro-F: CGGTTTATCTCCATGATTCGG***  ***MapGlcT4-1-pro-F: TCGCCTTCGTGTGGGGTTGA***  ***MapGlcT4-2-pro-F: GGATACAGGAGTCACCATAC*** | ***MaGWD1- pro-R: GTGTCCAACAGTATTGCTCATG***  ***MaPWD1-pro-R: TCGGCGGTGGAAGTGAAGAC***  ***MaSEX4-pro-R: GATGTGGTCGTATCACCCCCT***  ***MaLSF1-pro-R: TGGTGTGGGTCGGAGCTCGT***  ***MaLSF2-pro-R: GCGATTATTACGGGAGCCCTG***  ***MaBAM1-pro-R: GGCAGGGCGAGGAAGTACC***  ***MaBAM2-pro-R: ACTTACTGGGAGCATCACGA***  ***MaBAM3-pro-R: GCCATCGGGCTCGACGAT***  ***MaBAM4-pro-R: CCATCAATCCTGCCTCCATC***  ***MaBAM7-pro-R: AGCTGCAGCCAAAACGTGAAAG***  ***MaBAM8-pro-R: GAAGACTTGGAGGAGTAGAGG***  ***MaBAM10-pro-R: CGCCCTCATCGCCTTCTTGC***  ***MaAMY2B-pro-R: AACTGATAACTAGATGATTATCAG***  ***MaAMY2C-pro-R: AACTGATAACTAGATGATTATCAG***  ***MaAMY3-pro-R: CGGCGAACGGTGGGGGAGAA***  ***MaAMY3A-pro-R: AGAGTATCTGGGACTGAGCC***  ***MaAMY3C-pro-R: GAGGATGAGGAGGACGAAGG***  ***MaISA2-pro-R: CAGAAAAGTTCACACCTACGGC***  ***MaISA3-pro-R: TTGAGAGATCGAAGGAAACA***  ***MaPHS2-pro-R: GATGGGCGATAGCGGGAACC***  ***MaMEX1-pro-R: AGAGGCGAGAAGGTGGAAGC***  ***MaMEX2-pro-R: CAAGTTGAGACGAAGGAGGG***  ***MapGlcT2-1-pro-R: TCATCGCCTATCGGCTTCAG***  ***MapGlcT2-2-pro-R: AACTAAGGCCGGGCCGCATC***  ***MapGlcT4-1-pro-R: GGTACACCTCCTTTGGCAGC***  ***MapGlcT4-2-pro-R: GGGCCATCTCCACCCCGAAA*** | |  |
| **EMSA assay** | ***MaAP2a-R2-pGEX-F: ggttccgcgtggatccCGCCGCCAGAGTGGCAGCTT***  ***MaAP2a-R2-pGEX-R: agtcacgatgcggccgcTTATTCATTTGAGTAGAGCTTAGG***  ***MaGWD1-probe-F: ATGGTGTGTGGCCGCCATCATTCTCTCCCAAGCAACCACGTGTAAACAAAAATATCACG***  ***MaGWD1-probe-R: CGTGATATTTTTGTTTACACGTGGTTGCTTGGGAGAGAATGATGGCGGCCACACACCAT***  ***MaGWD1-mprobe-F: ATGGTGTGTGaaaaaaATCATTCTCTCCCAAGCAACCACGTGTAcacacaAATATCACG***  ***MaGWD1-mprobe-R: CGTGATATTtgtgtgTACACGTGGTTGCTTGGGAGAGAATGATttttttCACACACCAT***  ***MaPWD1-probe-F: GATTTGCAATATCTGAGAAATGCTTTATTTTGTTGCTTTGGCTGTCCAGGGACATTGTG***  ***MaPWD1-probe-R: CACAATGTCCCTGGACAGCCAAAGCAACAAAATAAAGCATTTCTCAGATATTGCAAATC***  ***MaPWD1-mprobe-F: GATTTGCAATATCTGAGAAATGCTTTATtgtgtgGCTTTGGCTGTCCAGGGACATTGTG***  ***MaPWD1-mprobe-R: CACAATGTCCCTGGACAGCCAAAGCcacacaATAAAGCATTTCTCAGATATTGCAAATC***  ***MaSEX4-probe-F: CTATGCTAACTCAATTAGAAGCTCATTGAGTTTGTTCTGGGATTGAGTTGGGCTCGTTA***  ***MaSEX4-probe-R: TAACGAGCCCAACTCAATCCCAGAACAAACTCAATGAGCTTCTAATTGAGTTAGCATAG***  ***MaSEX4-mprobe-F: CTATGCTAACTCAATTAGAAGCTCATTGAGtgtgtgCTGGGATTGAGTTGGGCTCGTTA***  ***MaSEX4-mprobe-R: TAACGAGCCCAACTCAATCCCAGcacacaCTCAATGAGCTTCTAATTGAGTTAGCATAG***  ***MaLSF1-probe-F: ACATAGATATTTTTTTCCGCTCATGAGAGGGCGGCGGCGCGCACTACTGGACGTTAGGT***  ***MaLSF1-probe-R: ACCTAACGTCCAGTAGTGCGCGCCGCCGCCCTCTCATGAGCGGAAAAAAATATCTATGT***  ***MaLSF1-mprobe-F: ACATAGATATTTTTTTCCGCTCATGAGAGttttttGGCGCGCACTACTGGACGTTAGGT***  ***MaLSF1-mprobe-R: ACCTAACGTCCAGTAGTGCGCaaaaaaGCCCTCTCATGAGCGGAAAAAAATATCTATGT***  ***MaBAM1-probe-F: TACCCCCCGCCTCCGGCCGCCGCCGGCACCCGTCTGCCCGCCGCCGACGCTGCCCGAG***  ***MaBAM1-probe-R: CTCGGGCAGCGTCGGCGGCGGGCAGACGGGTGCCGGCGGCGGCCGGAGGCGGGGGGTA***  ***MaBAM1-mprobe-F: TACCCCCCGCCTCCGaaaaaaGCCGGCACCCGTCTGCCCaaaaaaGACGCTGCCCGAG***  ***MaBAM1-mprobe-R: CTCGGGCAGCGTCttttttGGGCAGACGGGTGCCttttttGGCCGGAGGCGGGGGGTA***  ***MaBAM2-probe-F: CCTTGCGCTCCAATCGAACGGGACCGGCGGCTTGGTCATTGATTCCGGCACCACCTTT***  ***MaBAM2-probe-R: AAAGGTGGTGCCGGAATCAATGACCAAGCCGCCGGTCCCGTTCGATTGGAGCGCAAGG***  ***MaBAM2-mprobe-F: CCTTGCGCTCCAATCGAACGGGACCttttttTTGGTCATTGATTCCGGCACCACCTTT***  ***MaBAM2-mprobe-R: AAAGGTGGTGCCGGAATCAATGACCAAaaaaaaGGTCCCGTTCGATTGGAGCGCAAGG***  ***MaBAM3-probe-F: AAGGAAAAAAGAATCTAGAAAACAACAAACCCTAAGTCTCTTGAGAGTTTGGGGGTTCT***  ***MaBAM3-probe-R: AGAACCCCCAAACTCTCAAGAGACTTAGGGTTTGTTGTTTTCTAGATTCTTTTTTCCTT***  ***MaBAM3-mprobe-F: AAGGAAAAAAGAATCTAGAAAACcacacaCCCTAAGTCTCTTGAGAGTTTGGGGGTTCT***  ***MaBAM3-mprobe-R: AGAACCCCCAAACTCTCAAGAGACTTAGGGtgtgtgGTTTTCTAGATTCTTTTTTCCTT***  ***MaAMY2B-probe-F: ACCAAAAAGAAGTTCCTTGTTTTGTCTTTTTGTTGGCTAAATCTTTTGCATCTTTAGTT***  ***MaAMY2B-probe-R: AACTAAAGATGCAAAAGATTTAGCCAACAAAAAGACAAAACAAGGAACTTCTTTTTGGT***  ***MaAMY2B-mprobe-F: ACCAAAAAGAAGTTCCTTGTTTTGTCTTtgtgtgGGCTAAATCTTTTGCATCTTTAGTT***  ***MaAMY2B-mprobe-R: AACTAAAGATGCAAAAGATTTAGCCcacacaAAGACAAAACAAGGAACTTCTTTTTGGT***  ***MaAMY2C-probe-F: CGACATCACCTCTCTCTCTCTCGCTTTTTGTTCTTTTCATCATATTCGAATTCATTGGC***  ***MaAMY2C-probe-R: GCCAATGAATTCGAATATGATGAAAAGAACAAAAAGCGAGAGAGAGAGAGGTGATGTCG***  ***MaAMY2C-mprobe-F: CGACATCACCTCTCTCTCTCTCGCTTtgtgtgCTTTTCATCATATTCGAATTCATTGGC***  ***MaAMY2C-mprobe-R: GCCAATGAATTCGAATATGATGAAAAGcacacaAAGCGAGAGAGAGAGAGGTGATGTCG***  ***MaAMY3A-probe-F: TTTTTGTGGTGTATGGTGACTTGTTATTTTGTTTCTTTTCATGTAGATATCTGTTTATA***  ***MaAMY3A-probe-R: TATAAACAGATATCTACATGAAAAGAAACAAAATAACAAGTCACCATACACCACAAAAA***  ***MaAMY3A-mprobe-F: TTTTTGTGGTGTATGGTGACTTGTTATtgtgtgTCTTTTCATGTAGATATCTGTTTATA***  ***MaAMY3A-mprobe-R: TATAAACAGATATCTACATGAAAAGAcacacaATAACAAGTCACCATACACCACAAAAA***  ***MaAMY3C-probe-F: ACAAATAAATTATGGTGCCATGTTTATTTGTTTTTTTACAGGATAAATAATAAAGACGA***  ***MaAMY3C-probe-R: TCGTCTTTATTATTTATCCTGTAAAAAAACAAATAAACATGGCACCATAATTTATTTGT***  ***MaAMY3C-mprobe-F: ACAAATAAATTATGGTGCCATGTTTAtgtgtgTTTTTACAGGATAAATAATAAAGACGA***  ***MaAMY3C-mprobe-R: TCGTCTTTATTATTTATCCTGTAAAAAcacacaTAAACATGGCACCATAATTTATTTGT***  ***MaMEX1-probe-F: TACGTATATGTGTTGTGTATAATGGATTTGTTTGTAGATTGGATCTCCTCCAAGCTTTG***  ***MaMEX1-probe-R: CAAAGCTTGGAGGAGATCCAATCTACAAACAAATCCATTATACACAACACATATACGTA***  ***MaMEX1-mprobe-F: TACGTATATGTGTTGTGTATAATGGAtgtgtgTGTAGATTGGATCTCCTCCAAGCTTTG***  ***MaMEX1-mprobe-R: CAAAGCTTGGAGGAGATCCAATCTACAcacacaTCCATTATACACAACACATATACGTA***  ***MaMEX2-probe-F: TCTTTCTATTATTTCTTTCTAAAACATTTGTTTCCTTCGATTTTCTTATTCGGTTCTCT***  ***MaMEX2-probe-R: AGAGAACCGAATAAGAAAATCGAAGGAAACAAATGTTTTAGAAAGAAATAATAGAAAGA***  ***MaMEX2-mprobe-F: TCTTTCTATTATTTCTTTCTAAAACAtgtgtgTCCTTCGATTTTCTTATTCGGTTCTCT***  ***MaMEX2-mprobe-R: AGAGAACCGAATAAGAAAATCGAAGGAcacacaTGTTTTAGAAAGAAATAATAGAAAGA***  ***MapGlcT2-1-probe-F: CACGATATAATCCACAAGGCCTAGAACAAAGGCAACACTAAGAACACCGAACTGGAACA***  ***MapGlcT2-1-probe-R: TGTTCCAGTTCGGTGTTCTTAGTGTTGCCTTTGTTCTAGGCCTTGTGGATTATATCGTG***  ***MapGlcT2-1-mprobe-F: CACGATATAATCCACAAGGCCTAGcacacaGGCAACACTAAGAACACCGAACTGGAACA***  ***MapGlcT2-1-mprobe-R: TGTTCCAGTTCGGTGTTCTTAGTGTTGCCtgtgtgCTAGGCCTTGTGGATTATATCGTG***  ***MapGlcT2-2-probe-F: TTAGCACCGGCCGCGCTCCCTTTCCCGGTTTGTTCGGCGACGCGGGGGGAAGGGAGACC***  ***MapGlcT2-2-probe-R: GGTCTCCCTTCCCCCCGCGTCGCCGAACAAACCGGGAAAGGGAGCGCGGCCGGTGCTAA***  ***MapGlcT2-2-mprobe-F: TTAGCACCGGCCGCGCTCCCTTTCCCGGtgtgtgCGGCGACGCGGGGGGAAGGGAGACC***  ***MapGlcT2-2-mprobe-R: GGTCTCCCTTCCCCCCGCGTCGCCGcacacaCCGGGAAAGGGAGCGCGGCCGGTGCTAA*** | | | ***Bam*H I**  ***Not* I** |
| **CHIP assay** | ***MaAP2a-his-F: aatgggtcgcggatccATGGTCGGCGGGTGGGATCTCA***  ***MaGWD1-qchip-F: CGCCACCCCGTTATCACCTT***  ***MaPWD1-qchip-F: GTTACTACTGGCTCTTTGGG***  ***MaSEX4-qchip-F: ACCGAACGAGAGGACGATACG***  ***MaLSF1-qchip-F: TAAACGTAGCTCGATGGTGC***  ***MaBAM1-qchip-F: GCAAGAGTCCGCAGAAGAAC***  ***MaBAM2-qchip-F: CAGTGCTGGATTGTTGTGCT***  ***MaBAM3-qchip-F: GCAAGAGTCCGCAGAAGAAC***  ***MaAMY2B-qchip-F: AGCCGAGCGAGAAGAAGAAA***  ***MaAMY2C-qchip-F: CACACAGCGAGCCCTTGACT***  ***MaAMY3A-qchip-F: AGAATATGAGGGTCCGTGAA***  ***MaAMY3C-qchip-F: TGAAACGCAATAAAGCAGGC***  ***MaMEX1-qchip-F: GTTCTAACTATTTGAGACTTGGC***  ***MaMEX2-qchip-F: CTGCCGCCAGTGAGGTATTG***  ***MapGlcT2-1-qchip-F: CTGGAACAGCGGTGGAGTAG***  ***MapGlcT2-2-qchip-F: GACGTATCTGTGCCGTAAATC*** | | ***MaAP2a-his-R: gacggagctcgaattcTTATTTATCGCAGCTGCCAGG***  ***MaGWD1-qchip-R: TAAACCATCGCTCCTCACCC***  ***MaPWD1-qchip-R: CTTCACAACAAACTAAGACCAC***  ***MaSEX4-qchip-R: TGGAAGATTGTGTTTGAGTGG***  ***MaLSF1-qchip-R: CATACTCTCTTTCTCCTGTAG***  ***MaBAM1-qchip-R: CAAAGATCTAGCAAACGACG***  ***MaBAM2-qchip-R: GACAAGAGACCGATCACTAC***  ***MaBAM3-qchip-R: CAAAGATCTAGCAAACGACG***  ***MaAMY2B-qchip-R: GAGGCGAAACAAACCTAAGAA***  ***MaAMY2C-qchip-R: AGCCCATGTAAACAATCCTC***  ***MaAMY3A-qchip-R: TGGGCCAAAAATCAACGACTA***  ***MaAMY3C-qchip-R: TTGGTCGGGTCTTAGTGGTC***  ***MaMEX1-qchip-F: CACCTGCTATTCCTCCTTC***  ***MaMEX2-qchip-R: CAACATTCAGAAGTATCCCC***  ***MapGlcT2-1-qchip-R: GCCGTTATTTTCCTTGCGTT***  ***MaGlcT2-2-qchip-R: CTGTCACCTTCCCTGCTAAT*** | ***Bam*H I**  ***Eco*R I** |
| **Dual-luciferase transient expression assay** | ***MaAP2a-pEAQ-F: caaattcgcgaccggtATGGTCGGCGGGTGGGATCT***  ***MaAP2a-pEAQ-R: agttaaaggcctcgagTTATTTATCGCAGCTGCCAGG***  ***MaAP2a-pEAQ-PBD-F: tcgccgaccggtaggcctATGGTCGGCGGGTGGGATCT***  ***MaAP2a-pEAQ-PBD-R: aaccagagttaaaggcctTTATTTATCGCAGCTGCCAGG***  ***MaGWD1-pro-LUC-F: tatagggcgaattggCCGCCACCCCGTTATCACC***  ***MaGWD1-pro-LUC-R: ttggcgtcttccatggGAGTGACTCGCGGGAGAGAGAG***  ***MaPWD1-pro-LUC -F: tatagggcgaattggGTGGTCTTAGTTTGTTGTGAAG***  ***MaPWD1-pro-LUC -R: ttggcgtcttccatggCGCAGATTACGAGAGGGTTA***  ***MaSEX4-pro-LUC-F: tatagggcgaattggGATCAGATGTTATGGGAGGT***  ***MaSEX4-pro-LUC-R: ttggcgtcttccatggCCACATAGTGGGTGTGGCTG***  ***MaLSF1-pro-LUC-F: tatagggcgaattggGGCACTGATTTGAGTGCTGC***  ***MaLSF1-pro-LUC-R: ttggcgtcttccatggTGGTGTGGGTCGGAGCTCGT***  ***MaLSF2-pro-LUC-F: tatagggcgaattggCCGTCCGGGTTCCTTGAGG***  ***MaLSF2-pro-LUC-R: ttggcgtcttccatggCCCCGCCTCTTCCTTCTCTC***  ***MaBAM1-pro-LUC-F: tatagggcgaattggGAGAAACCTTGATGTTAGAGAG***  ***MaBAM1-pro-LUC-R: ttggcgtcttccatggCTCTTGCTAAATTTACTATCTT***  ***MaBAM2-pro-LUC-F: tatagggcgaattggACCTTCGGCGCAGCCGATCC***  ***MaBAM2-pro-LUC-R: ttggcgtcttccatggCTGCCGCCGCAGTTCTTCC***  ***MaBAM3-pro-LUC-F: tatagggcgaattggGCCTCCAGTACCTAACATTC***  ***MaBAM3-pro-LUC-R: ttggcgtcttccatggCGCGCCACTAAAAAGCCCT***  ***MaBAM4-pro-LUC-F: tatagggcgaattggTCGATCATGTCCCTTATCTC***  ***MaBAM4-pro-LUC-R: ttggcgtcttccatggCTCTCAAATTATAGAAGATGT***  ***MaBAM7-pro-LUC-F: tatagggcgaattggCAAGATTTATGGGAAGTGACG***  ***MaBAM7-pro-LUC-R: ttggcgtcttccatggAGCTGCAGCCAAAACGTGAA***  ***MaBAM8-pro-LUC-F: tatagggcgaattggGCTTGGAGCCCATGTCGTAG***  ***MaBAM8pro-LUC-R: ttggcgtcttccatggTCGAGTTGGTTCTCGAGGG***  ***MaBAM10-pro-LUC-F: tatagggcgaattggGCTCAAGATATCAGCTAAGAGG***  ***MaBAM10-pro-LUC-R: ttggcgtcttccatggTCGAGCGGGTTCTTGAGTCG***  ***MaAMY2B-pro-LUC-F: tatagggcgaattggCGTTTCCATGAGTGCCCGG***  ***MaAMY2B-pro-LUC-R: ttggcgtcttccatggAACTGATAACTAGATGATTATCAG***  ***MaAMY2C-pro-LUC-F: tatagggcgaattggCATCTAAACGATCCATCGCG***  ***MaAMY2C-pro-LUC-R: ttggcgtcttccatggAACTGATAACTAGATGATTATCAG***  ***MaAMY3-pro-LUC-F: tatagggcgaattggCGGAATCTGTTCTCCCGCAC***  ***MaAMY3-pro-LUC-R: ttggcgtcttccatggCGGCGAACGGTGGGGGAG***  ***MaAMY3A-pro-LUC-F: tatagggcgaattggGAGGGTCCGTGAAAAGGATA***  ***MaAMY3A-pro-LUC-R: ttggcgtcttccatggGATGATCTTTGATTGTTTCC***  ***MaAMY3C-pro-LUC-F: tatagggcgaattggGCACAAATAAATTATGGTGCC***  ***MaAMY3C-pro-LUC-R: ttggcgtcttccatggCTTCTCCTTGTCTAAACTCTTC***  ***MaISA2-pro-LUC-F: tatagggcgaattggCACCTGTGAGCAAGGACAAG***  ***MaISA2-pro-LUC-R: ttggcgtcttccatggAGAGCAGCCTTTCAGGCCTC***  ***MaISA3-pro-LUC-F: tatagggcgaattggGAGGCATGGATCCGAGGCTT***  ***MaISA3-pro-LUC-R: ttggcgtcttccatggTTGAGAGATCGAAGGAAACA***  ***MaPHS2-pro-LUC-F: tatagggcgaattggTGAGTGGGATGCTACCTGAC***  ***MaPHS2-pro-LUC-R: ttggcgtcttccatggGGGAAAGCTCCCTAAAGAATC***  ***MaMEX1-pro-LUC-F: tatagggcgaattggGGTGGAGAATATCTGGAGGT***  ***MaMEX1-pro-LUC-R: ttggcgtcttccatggTTTGGAGGGAGGGAGGCATG***  ***MaMEX2-pro-LUC-F: tatagggcgaattggGCCACATTGGATTCAAATGTC***  ***MaMEX2-pro-LUC-R: ttggcgtcttccatggCAATCAGAAGTCTAGAGAGAG***  ***MapGlcT2-1-pro-LUC-F: tatagggcgaattggCCTCAAAGATCTGAGAGCTC***  ***MapGlcT2-1-pro-LUC-R: ttggcgtcttccatggCGCCTATCGGCTTCAGATCC***  ***MapGlcT2-2-pro-LUC-F: tatagggcgaattggTGATTACAACAATCTTGAGCC***  ***MapGlcT2-2-pro-LUC-R: ttggcgtcttccatggCTCGATCGGCGCAGAATCTC***  ***MapGlcT4-1-pro-LUC-F: tatagggcgaattggGGCGGTGGTCGGAGGAGGAG***  ***MapGlcT4-1-pro-LUC-R: ttggcgtcttccatggCTTTCCCTTCTAATCCCCTC***  ***MapGlcT4-2-pro-LUC-F: tatagggcgaattggCGGCTTGTGGGTGCTCACG***  ***MapGlcT4-2-pro-LUC-R: ttggcgtcttccatggCGACAACCCGTTCGATCTTG*** | | | ***Age* I**  ***Xho* I**  ***Stu* I**  ***Stu* I**  ***Kpn* I**  ***Nco* I**  ***Kpn* I**  ***Nco* I**  ***Kpn* I**  ***Nco* I**  ***Kpn* I**  ***Nco* I**  ***Kpn* I**  ***Nco* I**  ***Kpn* I**  ***Nco* I**  ***Kpn* I**  ***Nco* I**  ***Kpn* I**  ***Nco* I**  ***Kpn* I**  ***Nco* I**  ***Kpn* I**  ***Nco* I**  ***Kpn* I**  ***Nco* I**  ***Kpn* I**  ***Nco* I**  ***Kpn* I**  ***Nco* I**  ***Kpn* I**  ***Nco* I**  ***Kpn* I**  ***Nco* I**  ***Kpn* I**  ***Nco* I**  ***Kpn* I**  ***Nco* I**  ***Kpn* I**  ***Nco* I**  ***Kpn* I**  ***Nco* I**  ***Kpn* I**  ***Nco* I**  ***Kpn* I**  ***Nco* I**  ***Kpn* I**  ***Nco* I**  ***Kpn* I**  ***Nco* I**  ***Kpn* I**  ***Nco* I**  ***Kpn* I**  ***Nco* I**  ***Kpn* I**  ***Nco* I** |
| **Y1H assay** | ***MaGWD1-AbA-F: ttgaattcgagctcgCCACCGCCACCCCGTTATCA***  ***MaGWD1-AbA-R: atgcctcgaggtcgacGAGTGACTCGCGGGAGAGAG***  ***MaAP2a-AD-F: ggaggccagtgaattcATGGTCGGCGGGTGGGATCT***  ***MaAP2a-AD-R: cgagctcgatggatccTTATTTATCGCAGCTGCCAGG*** | | | ***Kpn I***  ***Sal I***  ***EcoR I***  ***BamH I*** |

**Supplemental Table S2** Genes locus and names for publication.

| Protein abbreviation | Locus (NCBI, October 2016) | Locus (Genome, January 2016) | Locus (Genome, July 2012) | Publication |
| --- | --- | --- | --- | --- |
| **α-glucan water dikinase (GWD)** |  | | | |
| Glucan, water dikinase 1 (MaGWD1) | XP_009392285.1 | Ma03_p15660.1) | GSMUA_Achr3G14470_001 | MaGWD1 (Xiao *et al*., 2018; Fan *et al*., 2018; Liu *et al*., 2021) |
| Phosphoglucan, water dikinase (MaPWD1) | XP_009416602.1 | Ma09_p07100.1 | GSMUA_Achr9G07400_001 | MaPWD1 (Xiao *et al*., 2018; Fan *et al*., 2018 ) |
| **Phosphoglucan phosphatase** |  | | | |
| Starch excess 4 (MaSEX4) | XP_009413138.1 | Ma08_p13120.1 | GSMUA_Achr8T14150_001 | MaSEX4 (Xiao *et al*., 2018; Fan *et al*., 2018 ) |
| Like Sex Four 1 (MaLSF1) | XP_009416558.1 | Ma09_p06790.1 | GSMUA_Achr9T07030_001 | MaLSF1 (Xiao *et al*., 2018; Fan *et al*., 2018; Jiang *et al*., 2021) |
| Like Sex Four 2 (MaLSF2) | XP_009395158.1 | Ma04_p00190.1 | GSMUA_Achr4T00220_001 | MaLSF2 (Xiao *et al*., 2018; Fan *et al*., 2018; Jiang *et al*., 2021) |
| **Exo-amylase, β-amylase (BAM)** |  | | | |
| MaBAM1 | XP_009398630.1 | Ma01_p10710.2 | GSMUA_Achr1G17440_001 | MaBAM1 (Jourda *et al*., 2016; Xiao *et al*., 2018; Fan *et al*., 2018; Jiang *et al*., 2021) |
| MaBAM2 | XP_009420599.1 | Ma10_p10330.1 | GSMUA_Achr10G09320_001 | MaBAM12 (Jourda et al., 2016)\ MaBAM2 (Xiao *et al*., 2018; Fan *et al*., 2018; Jiang *et al*., 2021) |
| MaBAM3 | XP_009384530.1 | Ma02_p06550.1 | GSMUA_Achr2G04590_001 | MaBAM2 (Jourda et al., 2016)\ MaBAM3 (Xiao *et al*., 2018; Fan *et al*., 2018 ) |
| MaBAM4 | XP_009399963.1 | Ma05_p07800.1 | GSMUA_Achr5G08040_001 | Ma-bmy(Do Nascimento et al., 2006)\MaBAM7 (Jourda et al., 2016) \ MaBAM4 (Xiao *et al*., 2018; Fan *et al*., 2018; Jiang *et al*., 2021) |
| MaBAM7 | XP_009397011.1 | Ma04_p18390.1 | GSMUA_Achr4G17840_001 | MaBAM6 (Jourda et al., 2016)\(Gao et al., 2016)\ MaBAM7 (Xiao *et al*., 2018; Fan *et al*., 2018; Jiang *et al*., 2021) |
| MaBAM8 | XP_009403535.1 | Ma06_p07470.1 | GSMUA_Achr6G05840_001 | MaBAM9 (Jourda et al., 2016)\ MaBAM8 (Xiao *et al*., 2018; Fan *et al*., 2018; Jiang *et al*., 2021) |
| MaBAM10 | XP_009409087.1 | Ma07_p10880.1 | GSMUA_Achr7G11100_001 | MaBAM10 (Jourda et al., 2016; Xiao *et al*., 2018; Fan *et al*., 2018 ) |
| **Endo-amylase, α-amylase (AMY)** |  | | | |
| MaAMY2B | XP_009411354.1 | Ma04_p22000.1 | GSMUA_Achr1G02210_001 | MaAMY2 (Jourda et al., 2016)\ MaAMY2B (Xiao *et al*., 2018; Fan *et al*., 2018 ) |
| MaAMY2C | XP_009411975.1 | Ma04_p22020.1 | GSMUA_Achr1G02230_001 | MaAMY3 (Jourda et al., 2016)\ MaAMY2C (Xiao *et al*., 2018; Fan *et al*., 2018 ) |
| MaAMY3 | XP_009412382.1 | Ma08_p04100.1 | GSMUA_Achr8G04140_001 | MaAMY12 (Jourda et al., 2016)\(Gao et al., 2016)\ MaAMY3 (Xiao *et al*., 2018; Fan *et al*., 2018; Zhu *et al*., 2021; Jiang *et al*., 2021) |
| MaAMY3A | XP_009399983.1 | Ma05_p08000.1 | GSMUA_Achr7G26940_001 | MAmy(Junior et al., 2006) \MaAMY11 (Jourda et al., 2016)\(Gao et al., 2016)\ MaAMY3A (Xiao *et al*., 2018; Fan *et al*., 2018 ) |
| MaAMY3C | XP_009381896.1 | Ma10_p30040.1 | GSMUA_Achr10G30130_001 | MaAMY13 (Jourda et al., 2016)\ MaAMY3C (Xiao *et al*., 2018; Fan *et al*., 2018; Jiang *et al*., 2021) |
| **α-1,6-glucosidase Starch debranching enzyme (DBE)** |  | | | |
| Isoamylase 2 (MaISA2) | XP_009404709.1 | Ma06_p17710.1 | GSMUA_Achr6G16200_001 | MaDBE2 (Jourda et al., 2016)\ MaISA2 (Xiao *et al*., 2018; Fan *et al*., 2018; Zhu *et al*., 2021; Jiang *et al*., 2021 ) |
| Isoamylase 3 (MaISA3) | XP_009417596.1 | Ma09_p14380.2 | GSMUA_Achr9G14980_001 | Maisa (Bierhals et al., 2004)\MaDBE4 (Jourda et al., 2016) \ MaISA3 (Xiao *et al*., 2018; Fan *et al*., 2018; Zhu *et al*., 2021 ) |
| **α-glucan phosphorylase (PHS)** |  | | | |
| MaPHS2 | XP_009407534.1 | Ma06_p35790.1 | GSMUA_Achr6G33840_001 | Pho2 (Mainardi et al., 2006)\ MaPHS2 (Xiao *et al*., 2018; Fan *et al*., 2018; Jiang *et al*., 2021) |
| **Maltose transporter, Maltose Excess Protein** |  | | | |
| MaMEX1 | XP_009400667.1 | Ma01_p08940.1 | GSMUA_Achr1G15560_001 | MaMEX1 (Xiao *et al*., 2018; Fan *et al*., 2018 ) |
| MaMEX2 | XP_009397793.1 | Ma04_p34170.1 | GSMUA_Achr4G27620_001 | MaMEX2 (Xiao *et al*., 2018; Fan *et al*., 2018; Jiang *et al*., 2021) |
| **Glc transporter, plastidic Glucose Transporter** |  | | | |
| MapGlcT2-1 | XP_009421446.1 | Ma10_p15040.1 | GSMUA_Achr10G14930_001 | MapGlcT2-1 (Xiao *et al*., 2018; Fan *et al*., 2018; Jiang *et al*., 2021) |
| MapGlcT2-2 | XP_009413386.1 | Ma08_p19450.1 | GSMUA_Achr8G15970_001 | MapGlcT2-2 (Xiao *et al*., 2018; Fan *et al*., 2018 ) |
| MapGlcT4-1 | XP_009401350.1 | Ma05_p26070.1 | GSMUA_Achr5G23720_001 | MapGlcT4-1 (Xiao *et al*., 2018; Fan *et al*., 2018 ) |
| MapGlcT4-2 | XP_009380955.1 | Ma10_p25440.1 | GSMUA_Achr10G25480_001 | MapGlcT4-2 (Xiao *et al*., 2018; Fan *et al*., 2018 ) |

**Supplemental Text S1.** Nucleotide sequences of the promoter of starch degradation enzyme genes. GCC-box (GCCGCC or GGCGGC) and AT-rich motif (TTTGTT or AACAAA) are marked by green and red respectively. Translation start site (ATG) was shown in yellow box.

MaGWD1 (XP_009392286.1) (Ma03_p15660.1)

TCCACCGCCACCCCGTTATCACCTTTGTTTCTTTCTTTGGGTTGTGGGTCCGCCGCCCATGGGAATTTGCCCAAAGACTGGACCTTCCAACGACACCCCATCTCTCTATGCATTTAGAGAAGCTAAGTGCGAGGAATAACGAGCCTTGTTCTTGGAACAAGCGCCATTGCAGATGGGGTGAGGAGCGATGGTTTACATGCTAACTCAGATGTATCTCGGATTCGAATTTGTCGGATCAATTATTTTAATTAACCTGAATTTTCTTTTTTCCTGTCATAATTATCAAGTGGTCTGTATAAGGCACTCATTCGATATCGATAGATGTTCTTCTACCCTTCCCCCCACTTGCACAAATATCACGGATAGTCACAACCTTCCCGTGCCGTGCCTCCCACCCCTCGTATAACAATCTAATCACATGATGGTGTGTGGCCGCCATCATTCTCTCCCAAGCAACCACGTGTAAACAAAAATATCACGCTAAGACTCCTCTATTGTCGATCATCCTTACGGTGGGGCTCTCCTTAACTTACTCGTGTCCAGAACCAGCACCTCATCCTCACACACCAACACACCATTCTCTTCCACTACCAGCACACCACGTGGTAAATCCGGTTCACTCCGCCCTGGCATCCTGAGGTCGTCCAGGAACCAGGGATGTCCCTCCAACGAAAGTATGCTACGTGAGCCGTACCACCCGGTTCTAGTCACGTGCGTGATCGGGGCGTTGATCTTTTCCCCGCCTCTCTCTCTCTCTCTCTCTCTCTCTATCTCGCTCTCTTTCTCTCCCTCCTGCGAGTCACTCTGCGCCTCTCTCTCTCTCCCGCGAGTCACTCTGCGCCTCTCTCTCCTCCGTCGTCGTCGTCCTTCTTTTTCGGTTTCTTCCGCCAATCACCGCATCGCTTTCTTCTTTTCTCCTATTAATACCCTCCGAAGTAGAGCAAGCAAGAAGCTCTTCCTTCAGGAGGAACGTCTCTCCCTCTCTCCACTTCTCACACCCCCGCCCCCGACTCAGCGAATCGACATTCTTGGCGTGTCAAGGAAACCTTTCTTTTATTTGCGAAGGTAAATTTCTTAAGTGAATTCTTCGGGGTTCGAAAACCATAATCTCCTCCTCGTTTATTTATACCAGGAACAAAAATTCAGAGATCAAAAGGAAAAAATAATTAAGTCTCAAGAAAAATTGAGCTTATTTGTTTATGGCCTTCTTCGCAATAATTCTAGGGTTCGCCCTCGAGCATTATATAAATCTTAGTCACACTTCCATGACCATTCGTCCTCCTGCTTGATGCGGTCCCTGGAGCTGCAAAGTCCGATTTTAACGTTTCCTGCTCGTTCCGGTTCTTGGTTCCGCTGGACATGAAATAAAGCCGGGCCAGTAGTCTAGACAAAATTTTGATTAATCTTACTTTCTTCATGTTCTTTTTGTCGGAACTGGTGGGTTCAATTGTCAATCTCAACTTCTTGATATTTTTGAATTTTGCAATGCCAAGAAGATCCATGTTCGATCTGATCGGATGTCCTTAAGATTAATTAGCTTCCGTTGAGTTCGTTGATTATGACATTCTATCAAGTCCATGTACTCAAAGTTCAAATATGTGATTTCTAATTATGCTTAGGTCCCATGTCCCAGAATTCCTTACGGCCACAAACTGTTTCAGTAAAGTTTTTGTTTGCGCTTTATGAATATTTTCATATATCAGAATGGTCATGAATTTTTCGTGAGGACCAGAAATACAACTTTCTGATTGAATTATTGGTTTTGATGAGATCCTTCATAGCTTTCTCCCATGCAGGGTACCCTGCATAGACTTTGCATTCACCTGTCCTTTTTATTTCCTACTATATTATTTCTAGAACTAGCACCAAGGTAACTTCTAGTTCGTGTAAAGCGCTACATTCAATTTGAGCTTCAGTCTGCATTTAATTACCTTCTGAAGTTGTTATTGCTGGGTTTAAGAGTTGGTATATGATACAGGAACATG

MaPWD1 (XP_009416602.1) (Ma09_p07100.1)

CTACTGGCTCTTTGGGTAAAGTATTGTTACATTAGGAGTGTTAATTTCAACTACTTTTTTTTGTGGTCTTAGTTTGTTGTGAAGATTAAGAAATGCTTTATTCTGCTTCTAGAATTATTTCAAGGGCTATTTTTGGAGCTTTAGGTTGTATTGTTTTTGGAAGTAATATTATGGAGTTTCGTATACGGTTTCTAATAAGCAAAATTTTGGAATATAGTGACCAACAGATGCATAAATTACAGGAAGTTACTGTTGGTTTGATTAATGGCGCATACCTTGATATTGATAAATTTACCTGTATAGTAAGTGTTTTGAATCTTCCAAGTACTGATTTTTAATAGGTGCCTCATGCCATATGTTGCATAAATTTTGGTTGATTTGCAATATCTGAGAAATGCTTTATTTTGTTGCTTTGGCTGTCCAGGGACATTGTGTGTCCGGTTAAGTTGTAATTTGCCATAAATAATTGACACAAAAGTTACAAGTTAAGTTTGAATGAGTTCTTCAAGTTAATCGTGTAAAAACCTACTTTTGGTGAGTAACACAATTCAAAAGCAAATAGATAAAATTTGTGGACATATTTAACCAAAAATTGAAATGCTAAATTGAACTCTGTACAGCCAAATTAGAACTGGTAGGATGAATGATGGACCCTTTGTGCAATCCTATAATGCCAATTTACAATCGTATATGTGTTTTGCTCTATTAACTGCCAGATACAACTCAGCTCAGCTGACAGAAACCCAATATCGAAGTTCTTTGCAAAGAAAACTGATGATAAGGACCAGATGGAGGTAAAGCATGGTAAGTCCCTCAAGGAATCTCCGAAAAAAGAGATTTTTGATATAGCTGCGGAACTCAGTATATCATCTGAAGAATCGCCACAAGGAGATCACTTCGATGATCTGAAAGAACATCTGGAATTCAACACACATGCAAATGCTGATGAGTCTGATCACTTCTCCCTGCTCAAGAATCCTAGCATAGAACCAGAAATTTGTGGCACCAAAAGAGGCTCCGGGGCTATTGCACCTGATTCTGGACTAACATCTGAGAAGGGCTCTAAACCTAAAAAGAAGGCACGGCCTGTTAAGAATACTGGGGACAAGCAAGCTTCCCTCCTATCATATTTTGAGAAGGCCTAGACGTTTTTTTGTTCAGTATATTTTGATGCTTGAACATGTTCCTTGTACAACTGCAACAAGTTTGCTTGTAATTGTCCTTTTACCTGTGAAGTCCAAACACTAATCAGTGACCTGGTAACTATATACTACAAGTGTTCTTAGCTCACAAGAAGCCGGAAGTGTAAGCTTTTCTCGTGTGCTTTCATCTCTTGAAGCTTCTGTACTTTGTGCTGGAGCTTTCTTTTCTCTGTTCTCATGTCAAGCTTTCATGGTTACATGGTGTCACCTTTGCTTCTGATATTTCTGACTCTGTTTTGTAGACGAGCGTTACGCAGCGTTGTTTCGTTGTCTGGTGGCGGGCGGAATCAGCCGTGAGGGACCCGCACCGTTGTTCTTTTTCTTGCTGTCCGTCCATTGAAGCCATCTCGTTTCTTCAAGGATGAGCTCGGTGGCCTGTCATGTCCAATGCTATCAACTTGCGGAGGTAAGCTAACCCTCTCGTAATCTGCGATG

MaSEX4 (XP_009413138.1) (Ma08_p13120.1)

ATCAGATGTTATGGGAGGTCAATATGTCAATTGGGTAATATACCGAACGAGAGGACGATACGTTGAAAATTTAGACGAAGCGTCAGATGAACCAATGACATATCGTGTAACATAGGATTTGTATTTGTAATTGATTGTAACTATGCTAACTCAATTAGAAGCTCATTGAGTTTGTTCTGGGATTGAGTTGGGCTCGTTACGAGGCTCATTCAATGACTCAAAGTTAGGTCAGGTTATGGCACTGTCAGAAAAGACGATAGTACCACTCAAACACAATCTTCCAAACTGTGTTAAACAATGGTACCACCCAGATTGCAAAGTGGTACGAATGTTTTTAAGTTAAACATATTTTCTATATTGGTTTGATCGAACGAAGCTTTCAAATCATTGTAATTTTTACGAAGCACTAATTAATTTCTCTCCTTGGTGCCGATAAGATCATAATAGAATTATGATTGAAATAAAATTTAATTTTTTTATGATTAAAATTTGAAGTTGAAAAACATAATTAAGGAAATAAGCATTTTTAGGTGAAACTCCACACATACACAAATATATATATATATATATATATATATATATATACTATTTTTGTTGGTCTCATTCCGGTACTATCGTTTGATTATCAAGTAAAACATCTCTCATTAAAACATCTCACTATGATTATAAAAATAGATATTCACATATTTTCATAACTGAAATTAGTTAATCTTAAATCTTTACTAACAAAGATTAACCTGAATTATTTAGATGAATATTAATATTATATATATATATAATTTTAATATTTAGAGTGATACTTGCTATATCGAAATGTATAAGAACCCGCTTTTTGTTGTATCTTATTTTATTTGTGTTGACGAACCCTTCATGAAGTAATAGCCCGCAGAGAATCCGCATTCATCCATCCAAACATTTGTTTGGTTCAGCCACACCCACTATGTGGATG

MaLSF1 (XP_009416558.1) (Ma09_p06790.1)

GCGTGGGGTCTAATGCAGTCAAAATTATGCTTTTAAATTTCGTGAAGAGCTTATTAGGCACTTATTAAACACAAAAAATTATATTATACAAAGTTATTTTCTTAAGTATATAAAATATTCTTCTTTCGAATCGAAGTAGAATAAAATAAAGATATTTTGAGTCACGTTAGAACATAGGAATTCAATTGTACTTGACGTTTCAATCCAATGAAAGAAACTTGCACTAAAAAGCAATATATAAGGAATAGACTACAAACAGGAAATAAGGTGAACTGTTGTCATCAACCAAATACGTTAGCCATTGTGCATTATAATTTCATCAAAGGCAAATGAACATCTAAGCCACATTTAGAGAAGGAAAAAAAGGAGAGAAACTTCTGAAGCAAATGAGTCGCCGAGTGCTCGCCAGCTCGCACATCGCCCAACGAGATCACGACGCTGGAAAAGGTTTGTGAAGGCACTGATTTGAGTGCTGCATGATGACAACGATGAGGCCAATAATTTGATGTCCTGTTTGATTCCTATTTCGCTTCTAATTTAGAGATTATAATTCATCGATAAGGGCTTCGACGTGACACGTGTTTAAACTTAGATTTAATGCATGATTAATAGACAATTCCGAGAATATTTCATATAAACGTAGCTCGATGGTGCACTTGTCATATGTCATGATTAAGATGGATACTGCTATTTGATCACAAGATGCAAACAGTGCACTCAAACATAATAATCGTTCAAGTAGGCATCAATTACTACAGGAGAAAGAGAGTATGATAGAAAACGACCATACACTACTTTTACTAAATACCTCCAAATTGTTTGGAAAGCATTATATTAAAATATTCTTAATATATTTTAATTCATAAGGAATATATATATATATATATATATATATATATATATAGACTTTTTGTTATAAGGATGAAGAAGAAAATATATTCTGTTTCACTATATTATAAGGATTGTCTTTAGGACGAATTTTCAGAAAAAATATTCTCTTTTTAGAGTTTTCCGTATAAGTATCTAATATTATAATTTTCTCATAATAACTTGAAATATCATTTTTTTCTCCCTTTTTCTATGAACTAATATAGTCACAATGTTTTTACACTACGTTATAATATTTTCAGTAATATCAAAAAATACTATAATTAAATATTTTCTTATAAATTTAGTAAAACATTATGTATATTATTTTTGGGTAATAATTATGATATTTGTTATTATTACTAAAAATATTATAGCATAATATAAAAATAAGATCGGTTCACCCTATGTACTGTCCCATAAGAAAAAAAAAAAGGAAACAAAATAAAAGGAGATATATATTATGGAAAATAATAGTGTTAGGAAATTCTGAAAATTGGGATAGAAAAGTTCGGAAAGAAAATTTTAGAAATTCACACTTGTTTTCATATAATTATATATATTAAATAATATTAAACAGGTTTGAAGGAATAAAAATGTTATTTTGGAGATCTGAAAAGACGTGTGTGTGTGTGTGTGAGAGAGAGAGAGAGAGAGAGAGAGAGAGGTTAATTTATTTTAGAAAAAGAAAAATAAATCTAAACATAGATATTTTTTTCCGCTCATGAGAGGGCGGCGGCGCGCACTACTGGACGTTAGGTGGGGCCCACCGTGGCACGGTCTTCCCGTTGCGTCCCGTTCCGCCCCCTCCCGCTTGTCCGTCCACGTGGCTTGTGGATCAGAGACCGACGACGGTGTCGTCTCGTCCGCCCTCCGTCCCTCCCTCACCACGTCCAACGTAACCGACTCACGAGGAGGTACTGCGAAGAAGAACTCTTCTCCTCCTCCTCCTCCTCGCCCTGACGAGCTCCGACCCACACCAATG

MaLSF2 (XP_009395158.1) (Ma04_p00190.1)

CAGAACCCTGTGCTGATGGCCGATCCAACCTAAAATTACACGATTGTCAGCGAACACACTAAAATTACACGATTGTCGACGCAATTCCCAACTATTCGTATTACTTTGGTGGTAAGGGAAAGGGGTTCTTCTGGGGAGTCTTCTTCCTGGCGCTCTTCGTTGAGTTCCGGCTGGGGCTTGAGGTTTCTCAGCACGAAGAAACCAGCCAAGGTTGCTGACAAAAGGATGAGCAGCACTCTCAGGGGACACATCTTTCCACTTCTCTCTCGCTCTCTCTCCTCTTCCTTGAGATGGACGAACTCCGTCCGGGTTCCTTGAGGAGGATGAGAAAGAGGTTGCCAGCTCGACGGCCGCCTCCCAACACGAGTTCTTCTTTGTTTGACTTGGAACGAAACACCCATGGGAGGGTGAGTGCGGCCCACACAAAAGGAGTGGTTGGATATGGTGGGGCGTTAGGAGGACATGAGTCCGCAGGGGCCGAGTCGAATCTTGGCGGTGTCCAACCACAGCACTAAAAGAAAAGAAATGGATGTTCTTTCTAATGGGGGTTGAAGGAATGACGAGGGTTCCACCAAGAGACATCATGTGGCCAAAGGTGGGTCAATGGACTTTTTGTTTTAAGATGCAACTCGACATGCTTTGCTTTGCTTTGTAAACGAGCTGTGTTCTTCTCGACTCGACGACCCACGCAACACTCGATTTAAGAGTCTGAATCACTATTTTTTTTTTCTTTTCTAAAAAATAAAATAAAAATTAAATTTATAATAAATATATTTATTGAGTATAAATTATGTTGGATGAGGTTTCATAATTAAGTTTGAAAAGAGTTATTTATACAAGCTGGATGGAGCCTTTTTCCTTGGTCAATTATACGAATGAATGAATGGCTTGCTGATTCAAGTTGGATTGCATTTTGCATCAGAACAATATCTTTAAAGATAAAAAAGACTTCGACACTAGAAATATTAAAAAAGATTCAATGTAATGGCTCAAATCAATGGGCCTGTAGAATGGGCTCAAATCAATGGCTCAAATGAATGGGCCTGTAGGCAGCCCAACAAGCCATGTAATGGCGCCAAATGTTGTCCTGTCACGACGCCACCGTCAAGTTTCCCGCCAGAAAGGTTGCAAATATCACGTGCGGGTTAAGCCCGAAATATTAGAACTCGACGACATGTGCTCCAGAAAATTCTACCAAAAATATCTCTCTCATAATTATATTTCATATTATACTTATCGTTTGCACAACAACAAAAATAACAAATAAGATCGTAATTTTCCAGCTATTTAAATCTATATTCTTAACTAAAATAAAAATATTTAAATATTTATGATATGAAAATTTATATGCCCGACCCTTACTAATTGTTTCATATATATATATATTATGTGTGGTGGTTAAAATCAAATATAGAGATAAATTATGAGGGTGAATATACTATTAATAGTAGAATTATTTATCTTTGTTCAACTAAAAGGGTCTAATTGGTTACATTACATGGCTATAAAGTGACTTTACAACGTGGTCTCTTCCGAGGCGGAGGCGGCGGCGGAGGCAAAGGCGAAAGGGTCTGTGACGAGAGCGCGGCGATCGAGAGAGACAGAAAGAGAGAGAGAGAGAGAGAGAGGAGAGAAGGAAGAGGCGGGGATG

MaBAM1 (XP_009398630.1) (Ma01_p10710.2)

GAGAGAAACCTTGATGTTAGAGAGAGTCATAGAGAAAGAAAATTAGAAGAGAGAGAAACTCTAAAGAGAAAATATAAGGAGAGAGAAAATTCGAGGAGAGAAAATTCTTAAGGGAGAAAAATTACTAGATCACTATAACATTGATTAATTGGCAGAGAAAGATCAACATATTGTAAATTTGAGTACTTAAAAAAAAAAGGATTGAAAGCATGGATGATGATTTATTTTGAATATTATCACCTAGATTTAATGGGAAAAATATTAAAGATAAAAAAATATGATAAAAATTTTTCTTGGGTCAGAATGTTTAAAAGCAATTATCTTGAATAGTTTTAAAGATGTGATAAGCAATACATATTTTAAGTTATTCTTTGAAGAATAGAAAGAAAAGTTTATTTTTAACTAAGAAGAAAATAAAAAAGCATTGAAACTTTTCTCTTAATCAATACTAGATGAAAGTATTATTTCATGAATCTCTAAAACATGAAAAGAAACTTGGTTGATATTAGAAGATATTTATAAAAGAGGTATATATCCTTTCATGAAGATAAATTAGAAAAATTGTATCACATAGAATTTGAAGACAATGAAAATATCTCAATTAAAGATATTAGGATTAAACAAGATTTAGACTACGACTTAGATAATATTAATTTTCTTAGAGTAGATGATTTTAATTTAATTAGAAACATAAAAGTGGTTTTACCTCCATATATAAAAGATTTTACTCAGCTAGTAATTAAAGATTTAGAAGACGATAATTAAATTAAGAATTATCGAGCTAATTTTGAGGAAGAAGATAATTACAATAAATTTGAGCATAACTTTTGTATCTCATCAAATTGAAAAGTAAGAATTAGTGTAAACATTTCATCCATCATCTTTATTAAAAATTTTATAGGGTATTAGAGATTTCCTAATAATCTCAATTATAATGTCATTTAGTCCTATATTGGGCTATCCATTTAACACACACTATCACAACACGTTAAGTGCAAAAGAAGCTACCACGAAAGTCGCGCTCAAGCGTGCCTCACTGCCGCTGGCCCTCATGCCATCGGCGCGCTATCATCCATGCCATCTGCGTTACCACACGTGTCGCTGCCGGAGCCGTCGGATCCTCAGCCCCTCCCGACTCATGCGAAGGCGTCCGTGCCAATCATAAACCCAAACACGCGGCCGCACACGAGCGAAACCCTAGTTTCCCCCCATACCGCCCTCCCACGGTACCCCCCGCCTCCGGCCGCCGCCGGCACCCGTCTGCCCGCCGCCGACGCTGCCCGAGGCCCCGCTGGCGGAGCGTGTCGAGGGATCGCCTGCGTCCCGCGATCATCCGAGCCCAATCGCTAGAGGACGCCGGAGCCGCCTCGGAGCCCCGCCGGGGAAATCTGGTCTCGTCCTCTGCCGGAGTGGAGGAGGACCTCCAGCAGAAGGATCCGGTAGTGGGGAACGAGCTCTCAACACCTCCGATTCGTCTCCCTCTCACCGATTCGATGTATCAAGAAAAGGACTGTCTTTTCTGATTTGTTCCGCTGAATTTCGTGACTTTGGCTTGGTTAACCGAGATCCAGTCGCAAGAGTCCGCAGAAGAACTCAAATCCACGTGGGAGATGGCGAAATATTTTGGCAAATTCGCTCTTTTTGATTTCGTGGTACGGTTGTGCCCATGTTGTCCAAGCTTAGATCCGCCCAAAATGTAAGGGGCGTCGTTTGCTAGATCTTTGAAGCTCAGGGAAGGAGCAGAAGCAGATAAAGATAGTAAATTTAGCAAGAGATG

MaBAM2 (XP_009420599.1) (Ma10_p10330.1)

CGGTAGCGACCTGGTTTGGACCCAGTGCTTCTCTCAGCCCTCCGAGTCTCTCACCTACGCCGCTCTCCCCTGCACCAGTCCCCAGTGCCAGACTCTCCCTCGCTTCTCCTGCTCTCCTGATTGCCACTACAGCTACTCCTACGGCGATACTTCGTACACCAAAGGTGTTCTCGGCACCGAGACCTTCACCTTCGGCGCAGCCGATCCGGCGGCGATCACCGGCATCGCCTTCGGGTGCAGCACCGTGAGCGAGGTTGGAGCGGAGAACTCACCCTTCTTCTCTAACTCCGCCGGGATCCTGGGAATGGCGAGGGGGCCATTGTCACTGGTGTCCCAGCTCGGTGAAGAAAGATTCTCCTATTGCTTCGCTTCTGACGACACAACCACCGCTCTGCTCTTTGGCTCTTCGGCAAATCCGAGCCCACAGGCTTCTTCCACGCCGTTCGTCAACGTCCCTTCTCCCCTATACTACCTCTCCTTGCAAGGGATCTCGGTCGGTGCAACCCTCTTACCGATACCGAACACAACCCTTGCGCTCCAATCGAACGGGACCGGCGGCTTGGTCATTGATTCCGGCACCACCTTTACCCTGTTGACGGATCCCGCTCACGCGATGCTGAAGCAAGCGCTTGTGTCTCAGATCGATCTACCGGTGGCGACCGTGGCCGGGTATGATCTCTGCTTCTCCTTGCCACCGGATGCAAGTGGGGTGGCATTGCCGATCTTGGTTTTCCATTTGGATGGCGCTGATATGGCCTTCCCGGCGGCGAACTACTTCGTTGTGAATTCCAGTGCTGGATTGTTGTGCTTGGCAATCTTCGGGTCTCCGTTCAACCTCTCCATCTTGGGCAACTTCCAGCAGCAGAACATGCATCTCGTCTATGACCTCGCCGGTGGAAAGCTGTCGTTCGAGCCTGCGAACTGTAGTGATCGGTCTCTTGTCGATGTAGTAACAAAAAAGTGATGTAATTGCAATTATTATGGGTTTCATGACGTAAAACTGTCTCATATTTGTCGTAATGCAGTATTTTTAAGTTTATTTAATTACTAATAAAGGTTTTTTTGTTGTATTAAGTCCAATTATTCAGACTTAATATGTTTCATCAAATCAAATCATTGGTTGTTCATATCGTTCGTGCATTATTTAGTGATAGTTTGAGCCCTTCTCGAATTTGGAGGAAAGGAATATATGAGCTCGGAAAATTTTCTTGTTCGAACAATAATATGTGAACGATAAATTTTCATTTCTTTTAGAGAAAGAAAGAGAGACAAATCCAAGTTGAGATCATCCACACCACCCATTCTCTGTTGGGTGTTGGTAAGAGTCCATGTTGAGAACAGAGCAATTGCATCGAGTTCTCCGTGCTGCAAGAGCGAGCTATTCCTGATGACAAGCATCTCCTATGCAAGCCTACCTCATCTAGAGAGGTCATCGCCGGCGAGGACAGAGCTCCGCCTGTGGCACGGAGGGCATGAACTGGCTCCTGTGGGCGGATCGAGAAAGATGGATAAGATAGAATTTGGTGGGAGATTCTCCTCAGATGGACCTTTTAAGAGAGTTACTGCACCTCAAACCGTGACCTCTGCTTCTCCGACCATGGTTTCTGATCTTGAGGTACGGTTTAGTGCTACTGTCATGACAGTAAGAACTCGTCCATTCATGTCATTGCTGTAGATGTTCTGCTCGATATATACTCTGTTCATGTCTTTGTACTCAAGCCAAGCTAAGGATGGATCTGAGGCGCTTCCGCCACACTGAACTCCATGCAGACAGCAGACTCTCGCACAGACACGGAGAAGATGCTGGCAAACTACGTGCCGGTGTTCGTGATGCTCCCAGTAAGTTGTTCTTAGTTAATTGGCCCAGCAGCAGTGACATTACGGTCTTTGATCTGAGACTGCCCAACAGTTGGATGTCATCTCCGTCAGCAACGTGTTGGAGAAGCAGGAAGAACTGCGGCGGCAGATG

MaBAM3 (XP_009384530.1) (Ma02_p06550.1)

GCTGTTGTTCAGTCTCTCATAGACAATGGAATCGACGTAAAAGTTTATCTCGATTGATATTTTATGTGCTCGAGTACTCGATGACTGTTTTTCGGGTCAAAATTCCGAGAATTCCGGGTATCGATTTATCTTCAATGAGAATTTGGAGGTCCAATTTTTTGTTCTGAGTGTACTTATAATGGATTAAGGTAAGATGGAGAAAGTACCTTCGAGAGCTCAAGGTTTGTATCTGATGCCTACACAGCGAGATATTGGAGAAAATTTAAATTTCAACAGTAGTTAACAGTTAATCTCCACCAATTCTCACTTTCAAAACCCCAGATGCTGATGGCTGTGGACTATTATTGTTGAGCCACTGAATATAAGATATTAGAGGAAACTGAACCCTCCTTTTGATCGAGGTTCATGTTCCTCCAATTCTTACTTTCGGTGCCCTACATTGCTTATTCAAGTTTTTTGGCATGAACATTAGAGAATGTTGATCTGATTGTCTGATGTGTTTGATTGCCTCCAGTACCTAACATTCAGAAAAAGTCAACCAGGGGAATTTTCATCCATTGTTTTCTTTTCTTATTTTCTAAGGAGACTCATAAAAAGGGTAAGGAAAAAAGAATGTAGAAAACAACAAACCCTAAGTCTCTTGAGAGTTTGGGGGTCCTATCATCATCTACTCTGAAGAGGTCTTTGTGGGCTGTAGTTGACTTTGTTCTGTAAAACTGCTCCATTTATAGATGATTCTTCACAGAGAGAAGAAGTTCCATGATTCCGCTTTGCTACATACACCTTGCCCCATGCCAAGCTCATGACTTTGTCTGTCCTCTTTTGACTCTGGGTTGAAGGAGGTTGAAGTCACTTTTGATTGTAGATTGATACCAAACCTGGGATTCCCAGGAACGAGAATGAGAGGTTAACGCCACACGTAATGCTTTGTTTCCATGCAATACCGTAAGCAAGTTTTGCAGCACCAAATGCTTGACGAATTCATGCTTTCCTGACCAACAAATCATAATCCCACTAAGCAAGATTAAAATTATAACTACGTTACTGGAAAATTAAAGCACAAAATCTTCCCTCTTATATCATATTAAATACACATTTTCAAGTTCAAATCTACTATTCTGAGTGGAAAAAGATTGCCATTGCAGTCTTATTAATGCACTCTACCATATGGAATATTTAATCTTAAAAATTCTTCTCCAACCTAATAGAAAAAAAAAAAGAAGGAAAACATTCATATTTTCTACCTAGAAAATAATTTTAATTTCTTAATATAACTCTAAAAGTTAATTTTTAGAGCTAGAAAGCTCATGAAAAAGCAAAGAAAGACCCAATTCGAAAGGTTGAATTGTAGTTCATGAATAATTAATTGACCAAAGTTTCGAAAATCGAACAAAAAAGATTTTTAGTCAAAGAAACAGTAAACATCCATAGTCAATAAGTACCCTCAATCTAGTTAAGCCTCTATTTTTAGTCGGAGGACCCTCAATCTATTTTAAGTATCTTTCAATAACAATAACAAGTGGATTGATGGAAATGTGCGACAGTATATAGTGGTAAGGCGATCCTAAAATGAGGGCTTTTTAGTGGCGCGATG

MaBAM4 (XP_009399963.1) (Ma05_p07800.1)

TCAGCCTCTGAGTTCTCCACCTGACTATTCTATTTGACGTTATATTTAGCCATTGTTTGTTCTTTTGATAATGTCTAGTTATATATTTCTCCAATTCTGTTTATGTTGTTCATATTTAAGTGTCTCGAACATGCATTACAGTAATCATTTGATAAGTAAATTATTAGGCTTTCTTTCTTTTTTTTCCTGTGTTTTTTCTGTTCCTTTTAGTATCTTGGTCTGAATAATCTTTCTACACCATCAATAAGCTATGTATGACAGCTTGACCAAAATATATGAATGTAAGTTTTTGAGTTCCAATCATGATATAATTTCTATATTAAGGGAATTATGAGAACATTTATATTGGAATTTTTGCCATGACCTAAAGAAGCAACTCTATGATCTGATTCTTACTACCCACTATAATATTTTTTTCACTAAAATAGAAATTTGCTCATGCAATTTCAGTATGGGAATACAATGTAGAATCACAAAAGTAAACTGAGCTAAATGTTTACCCCAACAATTGTTAGGCATCCAATACATTTTTGCAAAATTTGTAAGTGACTGCATCTCTATTAGAAGTCTGATGTTACGTTCACATTCCTAGGAACAACACTCGATCATGTCCCTTATCTCAGGTCTAATGTATGAATCCTTGTCATATCATTCATGTGTTCATCAGAGAAATAATAGTGTTTCTTAAGATACATAGATTTTAAAGAACCATCACATTCTAATATGACACAGAAAATAACAAAATCTTGGACCGATAGGAAAGTATGGTTTTCCGTCACACCACACGCAAACTGAAGTGTTTGTCGTTGATGATTCTGCATCAGATTGGACGACGAGGGTTTTGGCACCACTTGTTATGTGGATAACTTGTACGCCTGAACCAAATCACCAAACTCGATGATCAAAATGCCAACCATGAAGAGAGAAGCAACCCTAATGCGGACGACCAAATTTGCTGTGCAAAATGCCGTGGATGCATCACGAACAGTTCCCTACCGAGTCCTACCTCGAATTGCGCGAGTGAGATGCCACCGACACGTCCACCTCCCCACATGGAGGAAGAGGAGAAGTCGCACTTGTCTTCCCCTCCTTCTCACCGGCCATTCACGTAATTTTTCGTCTCGGCAAGAACAGTAAGTTGTTCGATCTGGGAATCGATCTTTCCGTACACTTGGCTGGAATGGGACTCCTTGGTGGACCGAGTAAGGAGGAGAACGAATCGGTCAACCTGCGCCGCTTCCGTCGGAGCACCCGCGCGCCCCGTTCCGCGCCATCCTGCGGGTTCTCCCCACCGGCTGCCCTACCGGATCATACGAACCCGCGCGTCCGATTGGCATCGGTTGGATATCGCTGTACGGTGTTGAGAGCCATCAGGCTTCCTGAAGCTCCCGCTTCCCCTATCCTCAGCGCTAATACCCATAAAGTTGAAGAGAATCACCAGAAAGCCCCGCCACTTTCCATAGAGAAGGGGACTTCGCGGTGGGGGCATATGCGACATTTGGCATAACGAAAACGGGATTTGTGGATGAGGAAGACGAACCCACAGAAATTTGGAACATTGAGCACCGAGGTTCCGTTTATTTTGCGGCTGTAGATGAGCGTGAAGACGTGGCACGACCTTTTCGGCCTGCATTCTAATCTTCGCCTCCCTCTCCCCTATATAAGTGCCTCCCTCCTCCCTTCTGAAATCAAACCATCCCCAAATCCCAGCAGAAGCAGCTCAATCGACCGACTCCTCCTCCTCCTCCTCCTCCTCCTCTTGTTCTTCTCATTCTAATATAATTATCGCTTTTGGTGTGTACATCTTCTATAATTTGAGAGGATG

MaBAM7 (XP_009397011.1) (Ma04_p18390.1)

TAAATCAGTGAGAGTGAGAATCTTAAATTTTAATCTTGATATTTAGGATATATAGATTGATACTTCTTTATCATTTTATATTTAAAAATTTATTATTCTTCAAATCACTAACTTGATAAAGTGTTTGAAAGAACGTTTTGATTTATCTATAAGAATGAGATTATGATATAGGAATCAAAAGGATCCCTCACTGCTTTCAAGATTTATGGGAAGTGACGATTCAAAAAAATGGCATGATGAAATAAAAGATGAGTTTAAATCAATAGACCAAAATGATGTTTGAGAATTTATTAAATTACATAATAATTATAAAAAAATTTGTTATAAATGAGTTTTTAAAACTAAATATAACTTAAAGGGCAATATCGAATAATTAATTCCCTAAGGCCTTACATCTTAGAGTATTTAAAAGAATATGTTCTTATGATTAGATTTATGAGTAATTTATAAAAGATATAATGATATATGTTTTAATGGATAATAATTGATATATATATATATATATATATATATATATATATATAATGATTAAATATGATTGTCTCAATAAGATAAATTATAAGCATCATTTTAGACTATACTAGAAAGAATAAATAATATTATGGCACATAGAAGGAAGTAAGATATTGATGGCATATGAATGTTATGACTTATATTGATAGTGTAACTTAATATAATATTATTATATTTATTAGTCTTATTGGTCAATTTTATAAAATTTATATGCATATGTAAAATATATTTTTAAGAGTTTAGAATTTAATTGATTAAAATTATTTATAAATAATCTTTTAATTTATTTATTTTGATTTTTGATTTTTTATGTCTTATTAAACCAAGTGAAAGAACGTTAGATTATATGATTCTTTTTAATTATGTAAGATATATTATAGATTTGATTGAATTTTAATTATGATTATAAGCTAATAGAAAGAAAGTCAAATTATGATATAATTCCTTATAAGATGACCGAGGGGCTCTGATACTTATTTATTCTAAATTCTTTACTTTCACTTATAAATAAATAGGACCTATTATATATTAAATATTATAATATCATGTCCCCTTAGTCCTTATTTATTATTTATAGAAAGAAGTCTAATTGTACAAAGAATAGAAAGAAATGAGTCTAATTCTTCTTATAAATCAAAATTATTATTTCTTTCCAATCCGACGATCTACAAATAAGATATTCTGAATAATATCAAAAAGTACGTAATACTTATATTGATCTATCATTATTTGGTTTAGATTTTGATATTAAAATTTTTATATAATAAAAATATGATTATGCTTACGTGTCACTTTTTATATCCAACCATGTGATAAATGTGAGGCAGAAAAGCTACTCATGAGTTCCCTCCGATCACTTTTTTTCTACTTTTACATTGAAGATATTGAGCCCACCACATTTGTCTATTTCAAAGATTCAAACCTTCTTGGTTGATCTTTTTCTTTTATAAATCAGTAGCCTCTCTCAACATTATATCCTCAAACCTGTCTTCCTTCTCTCTCTCTCTCTCTCTCTCTCGTCTTAACAACATAATTCTGCACAGGATATAGTTAATTTTCGAGCTTTCACGTTTTGGCTGCAGCTATG

MaBAM8 (XP_009403535.1) (Ma06_p07470.1)

CTTGTTACACGACCACCAGCCACTTCCGCAGTGACTCGTAAGCCCTGCAGAATAAGAGTGAACAGTGTCGTCTTGTTGCGATGGTGTGACTTGCTTTGTATCTTTGAAGTTCGAAGCTCATTTCCTTGTGTATTGCATCGAGTCTTTTTAGTATCTCCACTGTTTTCTTCTTTGAAGTCAATGTTTTATCAAGTAGGTTTGCATGTTCTTCATGAAGGAAGTGTCCACTTTAGATGAACATAGCAGATCTAAACCAGACCAGTTTCGCCCGATCCAATCGGGTCTGAGTTGATCCGATTGAACCTAACTCACGCACGAATCTGCAATTCCTGTCGGGTTTTTGGGTGGGGTTGGATCATATCAAGAAACGTGTGCGTTCAAAATTCGAGATCGGGTGCGTCGGGTTCTCGGTAGGATTAGACCAGATCAAATATTATGCGGTCAAAGTTCACGTTGGGTTCAATGTAGGTCGAGCTTGGAGCCCATGTCGTAGTTCTGAATCCAAATCCTTCGTTCTCCAGCCTTGGCTAGTCTTTCCTTCTTCCTCCTTCTCTCAACTCATAGTGATACATATGATAGTACAATCAACAATGAGAAGACATACTTATCGATAATTTGGGTTTAAGAAATATTTTGTCCATGAATATTTCATTGAATATTCTCTTTTATTAACCCACTATAAAAAGATACCTAAATATGATAATCAAGATTAAATTTGTTTTATTCTCCTCGAGATTGACTTGAGTATCAGAAAGATCAAGTCGAAAAACCTATCTAACCTCAATCTTTGTCGAGGTCAATCATGATCGTTCCTTGGCCACTTTAACGATCAACTCAAATCGTGTCAAACAATTTATTTAAAAAAAAAATCTTCATCTCACTGTCATGTATTATGAACATGATTTTTAAGTTGTGAGCAAATAATAATGTGTTCTTTTATATGATGGAAGAAACAACGTAGATACATGACAAAACAACGCTATGGATAAAGAGAATTAAGAGAGTGGCCATCCCCATTTTTCTCATACTTATCTCATCGTTCTTCAACCTAAAGACTATTTTAGAGAAGCCTCATTTTAACTATGATTTGTACTGATCTGTCACTTCAGAACTGCATGAGAGAGAGAACGACTTGTTTGGAAGTCCATCAACCGGTGGTCTTCCAAATGTCACTCTCAACCCACTGACTGCTGAAGAAGATTTCTGAGGGACATTCTTCTAGACACCTCTCCGTCAAATTTGATCTCTTATGTTTGATGCTTTCTCTGAAGAACTTATATTTGCTTACAAATACAATGAAACACAATGTGTTTTGACTCATTTGAGATGAGCTCGGATTGACCAACTACATCTGGGATAATTGAAGAACAGTTTTAGATGCATGCAAGCAATAACATGGACTCCTATATCACCAGTAACAGGCAGTTCAGTCCTTGTAAATGGAGAAGAAGAAGAAGAAGAAGAAACTTCCATTTTGAGTACTGGACACTGCATCAGCCGAACTGTGGATCTCAAAGCCACACTGTCGTCTTATGAAGCAGAGCAACGCGGTTGACTCCCCTTATCGACCGATGTGGTGACCATGGGGTGCTTTCCCAGCTTGGTGGAGTAACGTAGGAGACGACGTGCAAGCGAGGATTTCCTGTCCTAATGTGCCCAAAGTTCAGGGTTGGACCGCTGACAATGCGACTAATATTCTGACACGTATAACATCAAATAAGTATTTTATTTTCCTCGTTGCGAAGGACCCTGTTCCCTGTCTGTGCGTGACAGAGAACAATTTAACTGGACCAATGTAAACGCTGTATGGCGAGTGCCACGTGGATTTTTTAAATTAACCAATAAAACTTTTTAAAGAGAAAAAAATAAGACAAAAAAGGTGGCTATAAATGTCTCTCCGTCAATGAGGACAACAGAAATGTGAAGTTGGAGAGAGATAGGGAGAGAGAGAGAGAGAGAGGAGGGCGAAAAGAACTCTTGCCAGATCATTGCTCCTCTCCCTCGAGAACCAACTCGAATG

MaBAM10 (XP_009409087.1) (Ma07_p10880.1)

GGGTGATCTGACTTTGGAACGAAGAGGTTGGATCGAGGAACGAGGGTCTCCCTACTGCAGGGTCATCTCATAGCGATAGGGTCGTCTTTTAGTTATCGCTAGTCCTACATAATAGGTCTATATTAGGGGCTTCTCGGCTCGATCTCTCAATTGTTTAAGCCAATAAGAGAATAGAGAGAGTATTTGTCTGCTTCTTGAGCTTGATTTAGGGTATGACTTTTATACCTATGAGCCGAGAAGTCTAGTCATGTATTAGCCAAGGCCCCCTTACTCTAAACGACAAATGCATTAATGAGGATAGTTTGCTGTCAGTCATTAATATAGTGGCATAGCTCAAGATATCAGCTAAGAGGTATCAAGTTGAGGCTTTTTGTTATTAATGTTCGCTGGTTTTATATCTTTATTGTTTATTAGTCCACCTAATGGAGAGAGCTATAAAGTATCAATATTATCATCCTTATCATTCAGGAAAGTTGAGGTATGCTATCATTAATGATCACCGATTTACGTGCCTTTATTATTTGTTAATCCAGGAGAGAGCTCAAGGGTATTAATATTATCCCTATCAACCATCCTCTCCAAAAGCCTAAGGGACAAAGGGCCTTGGTTGCTCAATCAACTCCCTCTAGATATTCGTCTTCAATTTTATAAGATAGATTAGTAATTAAAATATATTAAAAATGAGATTAGATAAGGAAAGTAACCGGGGATCAATCCTTACTTATAGTCTCCCTTTATTAGAAGTATTATGGAGGAATCGATCCCTTAGACGTTTCGAATGCCAACATTAAGTTATACGAAGAAAGAAGCTCAAATCCTTCTAAATATATAGTTATATTTACTTCACAAATGATGTCGTCTGGGACAACATACTCTTTTATTTGCTAAGCATTCTCTGAGAGATGATAAAAGATTGATTCTTTAAATTACCCTTAGAGTCAGGTTGTCAACCCAAGCTCATATATTATAATTAATACTTGCATTCATCACTTTTAAAAGGTTTTTATACTTGGTCACCTCCAACTATATTCCCATAGTTACTATAAATTTTCCCAATGTCAATAAACGAATTTAGACACCTAACATTAAAAAGGCAACCAACTAGGAAGTCGACTTATAAGTATTTCCTTTCATGCCATGCCATGCCATGCCATGCCATGCCAATTCTGACGCTTCTCGCATCGACTGGAATGTGTTTTGAAAAACTCATGCTACAACACTGCACGTTCTTTGTTTTTGCTGCAAGCTTCTACACAGGAGATGAGGAGGTGTTACTAAGGGTGTTGTAAGTCTACTAGAAAACAGATTGGGTTTTCGATTTTAGTTTTGTGAAACATGGCACGAGGACGACAGGCTCAACAAAGGAGATGGTAGAAATGCTAAATGCTGTCGTCGTCTTCTGAGTTAATAGAAAGCCTTTTTCTGTTTCGGGAAAAAATAAAATAAAAATAAAAATAAAAAGGCCCAATAAATTGGCCTCCATTAAAAAGGAGACGAAGCTCAAGAAGAGGCCAAAACAAACCTCACCAAATTACTTGTCCTCCCAAGACCCCTCCCCAGCGACTCAAGAACCCGCTCGAATG

MaAMY2B (XP_009411354.1) (Ma04_p22000.1)

CGTTTCCATGAGTGCCCGGTGATTAGGAGAATATTATTCTTCTTTTTTTTAAAAGCTACACGATGATTAGGGGAATTTTTCTTTTTTTCCATGTGCGCCAGGTGATTAGGAGAATTATTCTTTCTTTTTTTTTAACAAGATATACGGTGATTAGAGAAATTTTTCTTCCTTTCCATGAGCGCAGGGAATTATTCTTCCTTTATTTTGAAAAGCTACGCGGTGATTATGAGAATTTCTTCCTTTTCCATGAGCGCCGCTGCTTCGATTCCTCGACCCGCGGTCGAATTCGACCGGGACCATACGAAATCTCCGGTCAGAAAAGCGGGACCATTCGACCGGGACCATACGTAGGTCCCGCTTTTCTGACCAAAGAAGAGCTCGCGAAGAGTGGATGCTGTCTCGGCCCAGACGCGAAGGGCACGAAAAGCAGAGCGCGAGGGGGGCAAAAGAACAAACGCCTACCGGGCGCACAGGAAACCGATAAAGTTCTCCAAGGACGAAGACGGAAAGAGACGTGGGAGGTTTGATGTTTTCAGGGGAAAACAAAGGGGAGAAACGATCGCTTACGTCGCTCCTCGACGTCACCTCTCTCTCTCTCGCTTACGTCGCTCCTCGACGTCACCTCTCTCTCTCTCTCTCTCTCTTGTCTTTTCATCATATTCGAATTCAGTGGCTTGATGGTTCACAGCCGAGCGAGAAGAAGAAACGAGAGATATCGGAATCGGATCCATTTCACGCATTAAAATCGTTTTCTGCTGTCACGGACAATGATTCCCGAGCTGGAATTCGTCGTCCTTCACGCCTATAAAACGCTCGAGACAAGGCGACGGAGAGATCACAAGACGGCACACAGCGAGCCCTTGATTTGAATTGTTCTTAGGTTTGTTTCGCCTCCAGCGTCTTTGAGATGATGATTGAGTCGTTGCACCAAAAAGAAGTTCCTTGTTTTGTCTTTTTGTTGGCTAAATCTTTTGCATCTTTAGTTAGCTGTAGGTGCATTATATGGTCGTGGAGGCGATTGCTGGGAAACGTTTAGCTAAAACCCGTGAGACTCATTGCTTGGTATTGAGAAACGACATGCACTCTTTTAGCCCACAATACTGGAAAGAGTGCCTCAAATTCGAGGATTGTTTACATGGGCGTTTCAAGGTTTAAATGAGCTTATTCTAACCATAATTGTATGTGGTAAAAGTAAGGTTTGCCGTGCTAGGATCGTCATACCTATTATTTGAAATATCTGGCTGTGATATTTGCTATTTCATATTGCAAATTACTTAATGTTGTAAAGCAGAGTTCTGGAAGATGTTTTAGGAAAACTTAAATGCTATAGGAATATGTTGATTTTTGTTGCAATTTATGATATTGTTCTTCTAATTACTATTCTTCTTTTTCCAATCATTTCACTGTTGAAGTTTTATTCTGAGCTCAATGCATTACATTTATAAATGGCTGTCACTGCAGGGATAGAACCTCTCTGCATCTGCTTAACTGAATCAGAGGTCACAGTTATCTGATAATCATCTAGTTATCAGTTATG

MaAMY2C (XP_009411975.1) (Ma04_p22020.1)

CATCTAAACGATCCATCGCGTATGAAAATATATTCCCTTACCATATGTCAACATGAATCTTTAATTGACTATATAGCATAATAATAATAATAATAATAATAATAAATAGTAGTCCAATGGAATTGCCGACGGGATTGCCGACGTGCTTTGAAAAGGAGTACAATGGAATCGATGCCGTCGCGACGGTGCATGTAAGCTGTCAGCGGGGGAATGATTTGATCACGCATAGCCGAGATTGAGCTGTTCAAATGCGGCAGCCGTTTATGAACCATACGACACAAAAGCTACGCGGTGATTAGGGGCCATATGTAACATCCGGTTTGGATCGATGTGGATCCCGCTTTTCCAACCAAAGAAGAGCTCGCGAAGAGTGGATGCTGTCTGGGCCGGTCCTCGAAGTGGTGATGGGTCGACAATTTCTTATGTTATCGTTGTGAAGAAAAGGCCGGAGAGAGGTTGGGGCACAAAGCAGACGGCGACACGAACCGTGTCGCCAGCCATCTGCGCGTGCACGCCTCTTACTCTTCCGCCTTCACTTTGTCGACCCCGCGCGTGGGCCAAGATTCTGACGCACTCGTTGGTCGATTCCCAGCCGTTGGTGCCGAGAAGCGAAGGGAAAGACGATCAGAGCGCGAGGGGCGCACAAAGAACAAACGCCTACCGGGCGTACAGGGAACCCGATAAAGTTCTCCGAGGACGAAGACGGAAAGAGACGTGGGAGGTTTGTTTATGTAATGGTTTTAGGGAAATAGAAAGGGGAGAAACGATCGCGTACGATGCTCCTCGACATCACCTCTCTCTCTCTCGCTTTTTGTTCTTTTCATCATATTCGAATTCATTGGCCCTTCTTTCTTGATGGTTCACAGCCGAGCGACAGGAAAAAGAAAAGAGAGATCCCGGAATCGGATCCATTTCACGCATTAAAATCGTTTTCTGCTGTCACGGACAATGATTCCTGAGCTCGAATTCGTCGTCCTTGACGCCTATAAAACGCTCGAGACTAGGCGAGGGAGAGATCACAAAGACGGCACACAGCGAGCCCTTGACTTGAATTGTTCTTAGGTTTGTTTCGCCTCAAGCGTCTTTGAGATGATGATGGAGTCGTTGCACCAAAAAGAAGTTCCTTGTTTTGTCTTTTCGTTGGCTAAATCTTTTGCATCTTTAGTTAGCTGTAAGTGCATTATATGGTCGTGGAGGCGATTGCTTGGAAACGTTTAGCTAAAACCCGTGAGACTCATTGCTTGGTATTGAGAAACGACATGCACTCTTTTAGCCCACAATACTGGAAAGAGTGCCTCAAACTTGAGGATTGTTTACATGGGCTTTTCAAGGTTTAAATGAGCTTATTCTAACCATAATTGTATGTGGTAAAAGTAAGGTTTGCCGTGCTAGGATCGTCATACCTATTATTTGAAATATCTGGCTGTGATATTTGCTATTTCATATTGCAAATTACTTAATGTTGTAAAGCAGAGTTCTGGAAGATGTTTTAGGAAAACTTAAATGCTATAGGAATATGTTGATTTTTGTTGCAATTTATGATATTGTTCTTCTAATTACTATTCTTCTTTTGCCAATCATTTCACTGTTGAAGTTTTATTCTGAGCTCAATGCATTACATTTATAAATGGCTGTCACTGCAGGGATAGAACCTCTCTGCATCTGCTTAACTGAATCAGAGGTCACAGTTATCTGATAATCATCTAGTTATCAGTTATG

MaAMY3 (XP_009412382.1) (Ma08_p04100.1)

TGGGTTTGCTTCCGCTGGCCGTCGAATTCTACGACGACGTAGGGGCTGGAGGTGCCGTGGCCGTCCTTGGGGAGGAGGTCACGGGCGTCGATGACCTCGACGGCGAGCTTGCGGATGACCGGAAGCGGTAGGCTCGTCATGTTCTCATGTGAGGCAGAGGATCTCTTGCTTCTTGTTCTCTGATGAGGGAGGAGCAAAGGCGAAAGGAGAGGCGAGGATGCTCTCTCCTTTTGCTTTTGCTGCTGGATTCTTCTACCATGGATGGTTGGGTTGCTTGACATCGACGCTCCTCTCCATCCTGCTCTTGATTCCTTTCTTGCTTGTCGTTCTCCTTCTGCGTTAGCCGTTGGGTTGCTTCCCGCCCTTTTCCAAAGTGGATTCGGGACATAGAGAAGTCAATCTAATCCTCAACCAGTAAGGCAGGCAGCTGACCATGTCGACGCAGCGTTCATCGACTTCTCATGAGCTCTATACATTGGCGGTAGGTAATCTCGATGAAAACTGTCGCTGGTGGTGTGGTGGCGGTGGAAGACGGAATCTGTTCTCCCGCACTTTTATGATCATGTTTCCAGCCACTTGTGTTCCTCAAGCTGAGAGGAACAAAGGAATAAAGGCCACGATGGATGATTTGCCGCAGCAGCTTCTTGTGCTTCAAACGGTGGTCATTTGCTTCACGCCTTTTGGATCTCAGCCCATCGATAAGTCGACCAACATTTTTGGGCCAAGCTCAGATTGGTTGCCAACAATTAGATCCATCGCACAGCCCAATCGTCGAGCAACATCGGGCCATCCTCAACACAAGTCACTCACTACATGACATCAGCCTATCGCTCTCCCTTCATCCTTTTCCTCGCCCACCTTTTCACGGAACAAGCAAGTGGATCAGGTGGGCCAGTAGAGAGGACAGAGGCTTTGAGCCTATACGTTGCCAACTGGATTAGACATGTGAGGGGGTCCCAATTACACATGACAGGTCCTATCCAAATCCACTCCTCCATTTTTGTAGATCTCTTTCCACATGAAAGGATCGGCATTAATATCCAAAAAGATAAGAATGAATTCGTCTTCGCCTTTTTGACCTCTGTTTCTTCTCTTCATTGCCGATCCGGGCGTCCCTATCCTTTTCCAATGGTCACACTGGGAAATGCTGCTACATTTATCTTAGTTCTTATCATTATTGGAAGCATGTGTCTTGCATCCCTATCCCCCGTAGACGAATGAATCATTGTCTGAATCATCAATAGAAACCATCTATTTTATGGGAGATAGAGAAACTCCCGCGGTGGAATCACGGTGTCCCCGTTGGTTTCGCCACTGTGCTCCAGTTAGAAGCGGATCAAACTTTTCCTTATCCACGTGAAATGTGTCGTCGGTCGCCCGAGTCATGTCGAACGATTTCTTCGCTTGCGGACTCACGCCACACCACAGCCGCCCCTGACGCAATTAGCGTCGAACTCTTCCACGTAACCGCCGCTTTTGGCGGTACGTCGCAAGATCATACCCCCGCGAGCGCCGAGGGACTGTCGGATCCCGTTCGAGAACGCGTGTAGCCAGTGCTTCGATCCTCCAATGAATAGCCTCCGATCCGCCACGTGGGTCGTTTTTGAAACCTCCATCGAACCAGCTGATGTAGCAGATCCCGCTTCAGGTTGGCTATTAAAGGAGCCATGGCTCCGTATCTTCTCCACGTAATTGAATGAGGAGTGGAGGATATCCCCAACGCTGCTTTCATAGAGGCATCTTTTCCTGGTCGGTGTTGCGCTTCTTCTCCTGAAAAATCTCTTCGCTTCCTTCTCCTCCGTCTCTCGATTCTCCCCCACCGTTCGCCGATG

MaAMY3A (XP_009399983.1) (Ma05_p08000.1)

GAATTGGCATCTATCAGTGCCAAAGGCCTTTGGTTTCGGGGGATTATTATGCCATGTTACTGGAGCTGGTGGTTCTCACTGATGCTTTTCAGTTAAGAATATGAGGGTCCGTGAAAAGGATATGCATGTGATGCACATGTGGAGTGGAATCATTTGTAAAGCAAGAAATGACTAAGAGCAATTCATATCTTATCAATGTTACTATGTGATGCAAATAGTCGTTGATTTTTGGCCCACAAATCATCTCTTGTGTCGACCAAAAGAAAAATAACTCATATCTTTACGTCACGTAGAGATGCATGCATGCATTTGAAATTTGGCTTTCAGATGCATACATTCAATTCTAACACTCATTTGGCTTTCAGATGCATTCAATTCTAACACTCAAAAGAAGAGACTATGATTAATCATACCAATTGTAAACCAAAAAAAAGGAAATACTCTGATTATTGCGAAGAATTTGTATTTATTCGAAATTATTTTAAGAGGAGATGTTAGTGTATTGTTGGAATAAGCTAACACTAATAATCTTCATTAACGAATCAAAAGGGAAGCTTTCCGTAAGTACGTACTACTACTCAACCATGCATGCATGCATAAGGCCGATTGATACCATGCAATATACTAATCGTGTGACTTGAGAGAAAAAAAGGGGAGGGATTAAATAACATGGTTTATGTCTCCTCTCAGATGGCCTCATCGACGCCAATGATCAAAGTAATAATGCATCTCAATATCACGGTCGATTTCTCCATTATTGACTGCACCAACACTCAAACATTATGAATCTTATCTATACCTCTCTCTTACAACTCATGCTTTATTGTATATACACAACTTAATGTGTCTCAGACCCAATTTCCCAGTCAAGCTTCTCTATTTCTCTAAAGAAAGTACTCATCTCAGAATCAATAGGATGGGGCGGACAAATCACAAGATGAAAAGGAGTTCCCAGTCAAGCTTCTCGACTGCACATTATTTTGTCATGAAACCATGGAAAGGGACAGGAGAAACGTGCATGTAACAACATCTGAGAGGAGCACAAAATAATCACATCAAAGTGGAGGGAACGGGCGCCACCCAGCCCGTTGACCCATGAAACCAAGCTGGTTTTGAAGGCAATCATTGAAGAACTGAGTCGAACGAGGAGTAGGAACTCGATGTTCCTCCCATCAAATGTATGGAAGTGGAACGTCAGTGGTGTGGTTGTTGACTAGAACGAGAAAGAGGAGAAGCTTAAAAGAGATAGTTAAGAGCGTTTCTTTTAATTATGCATTTAGCTAGATCCAAACAGAAGAGCGGATCAGTTCATGTCTGCTGGGCCTGTCGATCTCGATCCGTAATCATCGAATGAAATGATCCGACCCGTTTAATTTGACTGGCGGAATTATGGGTCGAGTATACAGACCCGCTAGCAAGTCGGGTCGGATCTAAACACAACTTTAAACGTTTTAGGGGGGCCGAGGCAGTGAATAGGGTTTCTGTGAAATATTTAGCTTGTTTGGGGATCTAAACACAACTTTTAACGATTAGTTGGTCTTCATACTTGTGTCATTATCCTAAATTAGGTTTGCTGTGAAATATTTAGCTTGTTTGGGGAAACAATCAAAGATCATCATGCGAGGCTGTTTCTCGTTTGAATTGCGTGTGCTTGGTTTGATATGTTTTTGTGGTGTATGGTGACTTGTTATTTTGTTTCTTTTCATGTAGATATCTGTTTATAATTCGTTTGCATCCTCTTTTTTTTCAGATCTGAAAGTGAAATCACTTGGTCCTTGGATG

MaAMY3C (XP_009381896.1) (Ma10_p30040.1)

CGTTGACTGAGTTGTGCTACCCAAGCATTTATAATCCACCCATTTGGTATAATAGTCAATGATGACCGGAACTCGTTGACGTGTGAGATTTCCGTGTGTCAGTAAATTGGATCATCAAGCCACCGTTCTCAAAGGCTCGCTCGATCCAAAACTATAGTCGCCATTGTGCTGTTGGCCTTCTTCATGAGACTAACAATCGGTACTACTCATCTATCATGATACATGAAATCAATTAGGGATGGATTGCCTGAAGGAAGGAAGCCCACTACTGACTGAACTCGAGTCTCGGCTTGGTGCCACGATCTTCTGCCACTGGTTCTTAATTGGTCGTAATGTGTTCTTGGTGAGTGAGACCCGTAAAACGGGGAGTTGAGCATGCAGGGAATGGTGGAAGAACTTCCTCCAACAGTTGCTTGAAAGGCTTCTGCATTAAATCCATAATCTATCCGAGTAACAATTTGTCTTCTCGTGATGTAACGAATGACTCCATTTTTTTTCGCGTAGATTCCTTTTTTGGCTATGCACAAATAAATTATGGTGCCATGTTTATTTGTTTTTTTACAGGATAAATAATAAAGACGAAATTCGAATTCAGAATTTTTTTTGTGATAAATTGTTAAGATCAACTAAGTTAGCTGAAAATTTCTATTGATTAATGAACGATGTCACAGCTCACACTTCAACTGAGTGTAATATGATTGCATCGGTCAAAATTGCTTAGAGTTCATACAATCGAGTGAGTGTAGTAACTATGACATCTTATTAAGGTCTGTGCGTTCCTGTTCTTTAATACTTAAAATAACAATTTATAACGATATTATTTTTCCAAGATAATTACCCTAATTAAATATCTCAAATTCACACAATCAAATATAATATCTCAAATTCACATAATCAAATATAATTATAATAATTGTGGTTTTTTTAATTGGTACCCTCGATTTTATTGTTTTCAAATAATTACCCTGATTAAAAAAATAGTCATATTGCCCCTCAAAATTTTATTTTGACTTATTACAATGTTTTGATCATCACAATAAAAATATTATAAAGCTTACGTGAAAATAATATAAATAAGCTAAATAAAATCAAAACATAATAAAAATAATTTGATATATCAAACTAAGATTCAAATATGCATTTATAATATTTTATGAACAACATCCTCTAAATAGAAATAAAAAATTAGATTATTATAATGTTTTGACCATCATAATAAAAAAATTATAAGATGCACACGAAAACATAGTAAATAATTTAAATGGACTCGTAATCTCAACTAAACCAATTACAAGCTTAAAAATATGATGCTTATTATTAATGTCTACGAACAGTGATGGTGAAAAATAAAGTTAAATGATATTATAATATCACTTATAATAGTCTATGATATTTTCAATAAATGCCTATAAACACCATTGAAATTCACTATAAATAAGTTAAATGAAAATTTTATTATAACAATTTAAAATTATTAATAAATAAATAAAAATTTTGGTGAAATTTTTTTTTAGATGTATTTTGGATATTTTTTAAATTAGGGGTATTGTTTCAAAAAAAAAAAAAGCAAAAGATGGGATACCTTTTGGGCAATATCCAAAATCTGAATATTTTCTAGAAAATTATTCTTATAACATCTCATTCAAATATTTTATTATTATTATTTATTTATAGATTATAATTATGTATTGGGTTCTTAGGATGAATCAATATAATATATCCTCGTCTATAAGGATATGTTTGTCCGATCGTAATGATACTTTACATAAAAACACGTCCGGATAAATCGTCCGCAAATGTTGAGGTACGACTGCCATTACTGCAGCCTCCATTTGAGCCTTCCACAGACACGTCTGTTTCCGCCCCCTTCTCCGGGAAACTGAAACGCAATAAAGCAGGCGACGTCTATCCACAACTGTAACTGAGACGCTGCGGCTGCAAAAGTCTGTGTCACGTTGCCAACTGCCTTCTCCCCTTATCCTCGCTTCGGATTACCATTGGCGCTGGCTTCGATGATAGGGACTCGACCACTAAGACCCGACCAACCATGTATATAATGAATGGCCTTAGCTTTCTCATCGTAACCCAATCCGTCCCATTTTGTTCTGAAGAGTTTAGACAAGGAGAAGATG

MaISA2 (XP_009404709.1) (Ma06_p17710.1)

CAAGCATGGAACCAAAGAACTTAAGTTAATATAAGAAAGTATATATCTGAAAGTGAATCTTTAAGACTCTGACATGATCCCCACATCTTAGGTTCTTCAAAGATGGCTTAAATTAAATAGGAATGTTGCTTAAATTAAATAGGAATGTTGCTTAAATTTAGTCTAAATATCTTGAGGAAATTTGAAAGTTTAACCAAGGAAGAAAATCATCTTTGTCTCTTAAAAAAGGGATAAGGTGGCCTATGTAAGGCCATCTGGAAATCACTTCTCCACTGATCTCTCACTATCGTTATATCGTCTTTGCACATTGCATTAAATCTCAGAGAATAATAAGTATGATACAGAAGCTACATGCGCGCCAATTCATCAAAGCTTGATGTTTTATGAGATGGCTAAATTTCAGGCAGCCTACACTTTCGACGCGGGGCCCAACGCCGTCTTGATTGCACCAAACAGAAAATCCGCTGGTCTTCTGCTTCAGCGCCTGCTATTCTGCTTCCCGCCACCAGCAGACAATGAATTGACTAGGTCACTGGGTTTTCAGGTTCTACAACACCGATACATTGGTTTGATCTGATGTCAAATTTAATCGCGTATCTTCCTTGCAGTTATGTAATAGGAGACAAATCAATTCTGCATGAAGCTGGTCTACAGTCCATGAAAGATGTTGAAGCCCTGCCACCACCACCTGTGAGCAAGGACAAGTATCCATCTCAAAAGTTCCCCGGCAAGGTCAGCTATTTCATCTGCACCAGGCTTGGGAGTGGACCAAGGGTGCTCACTGATGAAAGTCTAGCTCTGCTAAGTCCCACTACTGGACTCCCAAAGTGAGTTTGCTTACAAGAGAACATGATGGTCATTGGAACTACTTTGTGAGAGATGCTCCCTTTATAATTCTTGGGTTTTGCCGGAATATCTTAGTATGATCCTTTTTGGATCGTTCCTCTTCTCTCTTTTCGCTTTGGGAAGAATCTATGCTTTTATTTTATGTGGGGATTGACGGACTTCTTGCACTAGCTATTTCTCTGCATATGGTGAGGTCAAGATATAAACAAGTTGCTCAGGTTTTGCTTTCGATTCTTGCCAAGATTGCTATTTATTGTTGTTGAGAATGTCTATGATGGCAAACGTAATCCAACTTGAATCGTATTTTCATTGGGAAGAAATATATAAGCCGTTGGTATATGATATATTATGCTATCTACTCGTGGCGGGTCCTGTGCGATCGAGAGTTGGGCGCGGACAGGGAACAGGGAATTAGATATGAGTGTAGCCCTCTGCTGGTCCGTGGTGTTCCAGTGAGCCGATGGGAAAAGGCTCCCTGTCATGAGTCAGACACGTGATCCTCGACCTTGTGGGTCCCACCGACGAAACGACCACCTTTCTTCGTTCACGTGATTCCCAGGTGCGTTGTCTTCCTCCTTGGAATCATCCCCATCCTCTTCTTAAATCAAAAAAATCCAGATTCTTATTTTGTGACCGAGAGAGCAAAACAGTCTCCGTCAAACATATCCCTCCTCCTCCTGCTTTGTTGCCTTCACCATCTCCCGTCCCTCAGACCGTTTCGCTTCGCCTCCATCTCCGTCCTCCGAAAGAGCCACGTAAAACCCCTTCCCCTCACCCTCCCCTCCACCCAATGCTTTCCCTTATTGATTAATCCCGAATCAGACTCATTTCTCACAGCAGGAGGCCTGAAAGGCTGCTCTATG

MaISA3 (XP_009417596.1) (Ma09_p14380.2)

GAGGCATGGATCCGAGGCTTGCAGAACATCGTTTTGGGTTTCAATGGCGTCGCCTGTGTGGGCATGGCCACATCCTTCCCCCTTGTCACAACGACTACTCCGGCTACGTGATGCCACGGACTTCTTTGTCGGGGATTGTTGCATGAAGTGCTTCTTGACGTGATCTGATCTGATGACCACAGTGATGACTGAGTTCTTCATGGCCGAGGGGAAAGGCGACAAGGACAAGTGGAATCCATGAGCTCATGGCAGGTGTTTGATAGGTGCTGCCACTGATTCCACAGTAAAGATACTTCTCATGCTTGCATTGTTACTGTGTGCAAAGGAAATAATCAACATGGACGTGTCCCAACTTTGTGGTTCGTCTGCTACTAAACTTATCAGGAACAAGAGGAGGAAGAAGAATCACAATAATTAATACAGGATTCTGACATGAAGAGGTTTAAGCCGAAGACCTTCCTTCTTACTGCTCAGCAGTAGTCTTAGAGGTTTGAGTCGAGAGTAACAGAGGTAGTGAACCGTGAATGCTACCATGAGTGTTGAGCTACTTAAACTACGTAGGGTGAAGGTGATGTGCATCATGGTTCATTGATTACTGAGCATTACTTGTTTGTACCATATAAATGCATCTATATTTTATGGATCGATGGTTCTAGTCGTGTCTATTGCATGAGATAGAAAACGACGTCATGACGCTGAATGAACTCTGGAAGCCATGTTTATATTTCTAAGGTCCTCTAGAAATTACATGGAAGGTAGGAACAACAATGTCCTCTCACAATGCTGGAGACCAGCTTCCAAATTACATCCAAAACAATTACTCTCCCTCTCTCTCTCTTAATCCCTATCTACGGGTTTAGGAAAGTGATAACCTGTAGCACGTGCTTGGCACGTGGGAGGAGTGGGACCCACGGACGCACTGGGAAGAATCATTCACCTTCCGATCACGTACGCGCGAGTCGAGACAGGTGGCCGCTCTCGCTGCTCTTTCATTGGTCAAATTTCACAGGGCCCCGCATTTCCCCATCCTTCACACTCGGGTAAGAACTGACTCCCGTTCACCCCCTGACGTCCGCAATCTTGATTGGTTCCCCTTATTGGGACCCACACGTCCCGAAGCGTTCACGAGACAAATGCCATGTTCTTTTAGAGAGAATTGCGTTTCACACGGAAATATTATACACACAAATGTACCCACATGATCATTACTGTAGGAATTTGCTGCCACTGAGATGGGTTTCTTTTCTATTGGTGTCTGAGCCACCGCCCGGTTCACGATAAACACAACCACATGAAGATGACAACGGAGATAACAGAAAGCAGGGAGGGGAGTCGTTGCGGGCCGCACATGTGTCGAGTTCCCCACATCCCGAGTCCGCTCACGGCGCAGGTGAAGTCGCTCATCTCTCAACGAAGACGAAAGATCACTATTAAGTGGCATTTTCGGTGATCGGAAGCCACCGTCTCTCTCCCCTTTCATCTTCTTTTTGTTTCCTTCGATCTCTCAATGCAGTGATCGGAATCCACCGTCTCTCTCCCTTTTTATCTTCTTTTTGTTTCCTTCGATCTCTCAAATG

MaPHS2 (XP_009407534.1) (Ma06_p35790.1)

CGTCATCAAGTTGGCCTTGTGTTTATATCATCCTTACATCGTTCTTAAGTCGATCACAGCTTTGTGTCGACTGCACCGTTCCTTCGAGTCAGCCGAAGAAGCTCGACTTGATTGAGTGGGATGCTACCTGACCTTTTCATGTTGGGAGTACGTCGGTTGTTGCAAGAAGATGACTGTAATTGGATGCCATTGAAACACAACCTGATGGTAAAATCTTTTCCTTATGGAGTTTTCCATCTCTATTACCAGACCGTACATGCTTTGACGTCAATATTAATAATTAAACTATAATAAGCGTTGACGGTCATGATCACACAAATTTGACCAGAGCCCGATCCACTTTGGTCGGGTCAACCTACGCCTAATCAAACCCGACCTATCTTGTTCGGGTCAGCCCATGACTGATTAAAATGCAGCCTACCATGATCAGGTCAGCCCAAACCCGACTCAAGCTTAACCGGACACGCACGATGTATCTATTCGCTTCCTCGGATACAATATATCCACTCAAGACATCAGCATATATAATCACACACGATCAATATGACAACTCTAATTGCAACGAACGGTTCATATCTCAAGCCGTTCAAGTCACGGTCCCCGTGTCAACTACCACCAGAAATAGCATTAGGTCCAATTTTTCACGTTTTGAGCATCCAAAATATCCTATTGTTCTTTTTTACCCTTTATCGGGAGGAAAAGAAAAATAAATATCTGATTCACTCGTTCACTCCTTGATTGAGAATAACAAAAGTTCCGTATTTTCTTTTTATGATTAGAGAAACTTCGATATTTTCATCATCCTTTTCCCCCCCCCCCCACCCCTCATTACTTCTCGATATATTTTTTTTCTTTTATCGAAGGCTTCTGCCCCTTCACAATACGCCAAAATCCGCCACTTTTGTCCGCTTCTCTCTGAAACCGTTCGTTCCCGGGACCCCACGTCTTTCGCCTTTTCATCGAGTCACGGACGCTGCACAAAAAATTCTCGCTTCCATCTCTTTTTTCTCCTCCTCGGGAAGCATTCTTTTTCCCCCTTCAATGCATTCTCATCTTGGATCCCCGGCTTCCCGCACGTGACTCACCGCCTAGGATTAGTAGGCTCTTTTGATTGAAGGCCGCGCCTTCGCGAAGAGAGGCGAACCCCATTCTCGAGTTTTTGTGGCCTCACTCGGCTGTCGTACACAGTGGTTACTTCTCTTCCAACTCAATTCTGTTTTCCTCTTCTTTTTTTCTCTCAGTTCGCGTGGTATGATTTTGATTGTTTATAATCCATTCCAACTTCATTGTGTTTTCTGATGATTCGAGTTCTTTATATCGTAGCCGGCCATTTCCTTCTTTCCTTTTTCGGGTCTTAATCCGTATTCGAAGCTTTTGTCGAAAAGCAAAAGACAAGAAATACTTTAGAACAGCTAAAATTATAATTGAGATACATATTGCTGAGCTCACATGTCGTGGTTATGGATTCTTTAGGGAGCTTTCCCATG

MaMEX1 (XP_009400667.1) (Ma01_p08940.1)

AGAGGTCAAGGTGGTGCTTGTGGTCCAGTGCAAATCTCTCTGGTGGAGAATATCTGGAGGTAGCAGCTTCAAGAAACAACAGCAAGTCACATTTAAAGGTGATTTCCTTTTTCAGAACAGCAATTATAAAGTTCTAACTCTAATACCCTTAATTAAATCAAAATCATCTAAATATTTATTTCTAGTTTGATACTTTTGGCCTTTAATGATGTCACACTTTGTTCTGTGAAGGAGGAATAGCAGGTGATTCTTGTTCATAGCTATTACAGATGTTAAGAGCATCAGTTTCCTCTTTTCAAATGGTGAATAATAGAAGCTCTTTTTCTGCTTAATTCTACAACAATTTTAGGGCACTGCCAATTGGAGGCGATCAAACTCAGTTCAATGGCTCGATCATTGCTGATAACGGAAGAGGTTGTGGAATGTTGTAATTGGTATATTTTTTTCCCCCACCTTTCAATTACTGAAACAAATCCTTGTGTCAATGACCATGTTAAGCTTTGAGATAATTTGTTTGTTTTTGTATCTATAAACTGCAGGTGAAGGCTGTGAAGTGAATGACAATTAGCATCTCTCTGCTAAGGTATAATCCCATTCTTGATAATGCTTGGAACTGCCCACTGCCTCCAAATATGAAAAGAAAACATTAAAAAAATGTATTTTATTTTTCCATACATGAGTATTTTACACTTATAATGAGACTTAAATCATCAATCATATAACCATCCATCCCAAGACAAAATAAAAAGAAATAAAAATTATATTCCTTCCATAGATAATGATAATGATAATGATAATAATAATATGTGCATGTGGAGGACAAGCTCTTCAATCCTCTCTTGAAACCTCCTAAAGCCGGAACACAAGCTCGGCCACCACAAAGGCTCCTCTCAATCTCATCCAATATCCGTCCTGTCACTATAGCAATTTAGGTGAATTAGGGATTCACAAAATGTGCCCCTATTTGAGAATTTTCAAAAGAGCATAACTTGCATTTCAATTCTTTATATAGTTTCTTTTCTTATTTTTCTGAAGTTGAAAGGCTATTTCATTATTTTATACATAATTTTGTAATATCCTTTTTATATAAATTATAATATTTTATAGTAAGTTTCAAACTCCACTCTGTTCTTAAAAAGATTTTACATACTCAATGACACAAAAAAAAATGTTATTTAAAAATTTTACAATAAATTTGAGCATAAATACTAATGCATACCATAAATGTACCTATATGTGTTGTCTATAATGGATTTGTTTGTAGATTGGATCTCCTCCAAGCTTTGCACTGCTCCCATCTCAACCGTTCATCCATTATCTCTTTTCTCCAGGTATGGGTCCACCATGGATGAACAGTTGAGATGGGAATTGTACGATTTTATTTACCCTGCATGAATTGGAGAGCATCTGCCACGGAGTAGATTCTCCTCATCCCGTCTCGCTCCTCTCTTTCCCTCGGTTCTCCTCCCCATGCCTCCCTCCCTCCAAAATG

MaMEX2 (XP_009397793.1) (Ma04_p34170.1)

GCGTGGAGTTGATATGGGTGACAATACTTTCAACGTAAGCATCACTGTTCTTACTGTTCTGCTTAACATGTTTAATACCATGTTTCTTCTTCTTGTTAATGGTCATGACATAGCATATATTTTTCTTGATATTCTAGTTATTCAAATGAGAAGTCTGAAGCAAGAAATTCTGATGCCACATTGGATTCAAATGTCAAATCAGTGTCTGCCGCCAGTGAGGTATTGCAATCTATAGTTTCTTCGAAGCTTGTTGAAGTCATCATACAGATGGTCAGGGGATACTTCTGAATGCTTTTAATCTTTCCCATATAATATTTGCAGGAATCAACCAAATAATTCATACAAGCTAGCTGTTGGATTGGCATGAAATATCGCTAATCGGAAATGTAACCGAAGCTGCATCAGGATCATAAAACGCATGTACAAATTGTGTATGTGGTAGGTAAATCGATAGTACAGCCACATGGTGGGAAGATAGTGTTTGCTTTCCATGCTTGTAACCGGTGACACTAGTGCCGATTCTTGCATATGTTGCACAAGTTTTGCAATGGGTTCGATTTTTTTTCCAATTATTCTCTATCATTTATTATTTATTCACTTGCATGAATGCCGACCGAAGATGTTAAAAACCACCTTATTTATTGGCCATATAATCGCATATCTTCATATTTCTGTTTAAAAATCAGAGTGAACCGGCTGATTTATTCACTCCTGGTTTTCGTATAACATCATAAATCAAATAAAATCATTTGGTTTACATGATATTCTTAAACTTGCTCAAATGTCATATAATTCGATCTTTCTATTATTTCTTTCTAAAACATTTGTTTCCTTCGATTTTCTTATTCGGTTCTCTCCCTCTTATTTTTCGCTTGTTGCTCCTCTCCCTTCAACCCTTCTTCATTCTTATTCTGATGGGATCTTCTCCCATTCGGTTCCTCTTTTTTCCTTTCAAGCTTCTTTAAGTACAAGTAAGGTTCTCTTTTTTATCAGAACAAGAATGTTTCTATCAAAGTCATTCTTCTCCTTCTGACAAGATCTTCCTCCTACAATGTTACTCTTTTTCCTCTCATACTTCTTTTATCTGACAGTAAGACTCTTTTGTTCACAATAAGAAAGAGAGTGTCAATATATTGATACAAATACAAGTATAGGAGAGATCAAGATATAAAAAATCATAATTCAATTAAGCTTATCATTCTAAATATAGTTATCATGATTGAATTGAAGAACGATTCGATGGAGCCTAAGATGAATAGATCTGACTGGATAAGTGGATTAATTGGTTGAACCGTATGCTTAATATAATATATATAATTTAGAATTATATATTAATATAAAACTAAAAAACAATTATTTTTAAACATAAATGGCCACAAAATATCAATCATACAAAATAAGTTAAAAAGAATATTTTGAAAAATAAATTATATTGATCCAATATAATAGTTAGGAGAAGACCATATTTATATCTATCTATAAACTAGATCTTACTTGACTAACCTTATCAAATAGACATACACCGTAGAGATAAGAAGGGCAGAGACTCGAGTAATAAACGCCGTGTTGGGATCCGAGCCCTCGGTAATCCCCTTCTTTGCCTCCCCCCGCTGGTCCGTCGCCGTTAACATCTTACTTCTGTAACCGTCCATTCCCTTTTCCCACGCGGACCCACCCCACGTCACTCTCCATCCCGTTCCGCGTCACGTGGCTCTTCTCGTGCCGTCGGTCACCCTGCTCTTCTCGTGCTTGGAGGAAGAAGGCCCCGATGTCCTCCCTGCTTCCCATCTCGTAACTCCTTCCCTCGCCTACCTTCCTCTCTCTCTCTCTCTCTCTAGACTTCTGATTGATG

MapGlcT2-1 (XP_009421446.1) (Ma10_p15040.1)

CAAGATATCCTTAGTACCATGCAGCATATCAGGAACTGTTAGTATGTGTTTGTGCATTTTGTATTTCTTCAACGGCCTTACAATTTCGATTGTTATTTTTGCTGTATGATTTTTCAATTGTTGATACATTTCAGAAATAAAGTGCTAATTGAACCAATTAATTTTTTGATAAAATTATTGTCAAACTATGATTCCATTGTTCTATATTCTCTGATATTATTTCTAATTTTCCTCCATCAGAGAGCTGGAGCTGTATTTCCTCAAAGATCTGAGAGCTCTGCACTTGTATTTACGCCACAAACATGGCCTCTGAGATCCTATCCTCCGTCAGTACGAAGTCCTGATTATCAAAATGAAACACCTGAATCTTCAATAGCATCTGATTTTCCTGCTTTGAGGTGTTTCCCTATTCTCATCAGTTATCTGCAATGAACTTCATCACACCTAAGTTTTGGTTCTGATACAAAATTTATTTAATTTTTCAGTCTCACATAGATTCAGAATGCCAATGGTATATTTGGATTCTCGGTTCTTAATATTTGGGATAATCAATATAAGCATATTTTAGAATTTTTCATTTCTATTTAACTTTAATTCAAACGTAATGATAAAAAGTTAGTTCATGTCCTATCGTACCTAACACTTCTCGTCTTTTTTGACCACTGCAATATAAGTATTTTGATGACTATTTAAAATTTTATTTAATATATATTAAATATTAGTTTGATGGTATAACAGACTAATTTACCAAAGTCAGAGTTCAAAAGCTCATTTAATATATTTTTATATCTTAAATATTTTTAAATATTAATTTAAATATAATATATTTTTTAAAATATTAAATTATTTTAAATATTAATTATAAATATATTTTAAATATTTAATTATAATTTTTTTATATTTTAAATATTAATTTATATCTTAAAATATTTTAAAATATTTTATATTTTAAATATTAAATTAATTATAAAAAATATATATTAAAGTTATTTATAAAATTTTAACAGTTCGAAATTTCACGATATAATCCACAAGGCCTAGAACAAAGGCAACACTAAGAACACCGAACTGGAACAGCGGTGGAGTAGGTTAATTAAACTAGTTATTCACGAGTACCACCGTCCGCGCCGCTTCCACTATGCGACAAACCACTTTGCCGGCCTTTTACACGCGTCCGGCTGTGGACGCGGCAGGCGACCTCAGTGACGTCGCCTCGTCCACACGCCTCGCGGCGCGCGGCGGACGGCGCAGGTCACGCTGTCGGTCGCCCACGTTGCCTTACGTTACGCACGCCTTTTAACGCAAGGAAAATAACGGCCGTTACGCCCCTTCTACCTCTCTCCCGCCTTTTCTAGATGTGCGAAGAATACTTGTAAGCCAGAAACTTGATGCTTAACGGAACGTTATAAACTTTATTATAACGGTTTTGGGGTGTCCGTTACACCGGCCAATAAATAAGCGTACCAGATCTATATGTTGGCTTTAAGGTGTCGCCAAACCCTTTCTTTTTTAACCACAAAAGAACGTAGAGGGATCGAAGGCCAATCTGTTACCTCTGTTATCTCTCGAGCTGGATACGATTCGATTCACTTCGATTCATCTGTCTTGTAACGATTGATGCGATCTTGATTGCTTGATCTCCAAGAATAAGCGCTGGAGAACGGGGGATCTGAAGCCGATAGGCGATG

MapGlcT2-2 (XP_009413386.1) (Ma08_p19450.1)

CGGTTTATCTCCATGATTCGGATCGATGACCGACTTATGAACTAATAACACTTGAATTTAAATCCTCCAAATTAGATTACGGTAATTTGCAGGGCAATTAAGTCTCATTTTGCTCAGCTACTCGTCACAAGAACTACTCTTTTTCTTGCTGTCACAAAGAAACACTCAAATTATGAGTACTGTAGATTAGAAAAAAAAAACATCTTTTAATGGCCAAAAATTAGTGATGAATTATGGGCAGGATGATTAAACATCATGTATGGTGTTTGACATGTTCAAAGTTTAATGCATCAAAAGAAGCTTGGTCATAACTAATAAAAAAGACCTTATCCCCTTGATTCAGAAGCTTTGCTCGAACCATGTTTCCTAGCATTACAATCATTACTCTTTATTTCTGGAACTATGATTACAACAATCTTGAGCCATTTTCTGCATAATTTATTCATCTCTAACAAATAACATAAGTAAAATCTTTTTTTTTTTTTTTTTTGCGTTCCACATAAGTTGTTAAGGGAACGGAGCCGAGTAGAATGGTAATATTACTCTTTTACAACCTTAGGGATTATTATTTAATTAATACGATTCGTGTGTCTCGGATATATTTTATTTTTTTAAGTTGATGAAATAAATGAAATATATAAACAAGAGATTTCAAATAAAAGATCACTTATGTTTTTTGAAACTTAGGCGGTATAATTGTTTGAGTATTTGATGAAGCATTTATATATACATACATTCATATACATATATATACAAGCATTTCACATAAAAATTATTTATGCTTTTCTAAGCCTTATAAGCTTGCATTTTATACGAAGAGTCACCTCAAAGCGACGCCTCTACTCGTGAAACCAATTACTCAACCGATGTTAGTAGTCCGTTTGAATTCACCCCAATTTGTACAGATGCACTCCTTATCTCTCATGTTGCCAGCAGAGATCAGGGCGGTGCACGAGCGACGGACATGGGGTGAGTTTATCACTCGCTTGAGTTCGTTACCACCGCTCACCACTACGCGACCAACTGCTTCCCCGCGCACGAGATCCCACTTTTCCTTACCAACACGTGGCGGACGATAAAGCATCAAGCTACCGCAATGACGTCCGCGGTTGGTCACGTTATGTTGTGATATTTAACGAACACATGAGATTGAGATCACACCGACGGGACTTGCTGACGTATCTGTGCCGTAAATCAATACTTTAAAGATCGTGTCACAACAAATCAAGAACTTCCCATTATGATTTGAAGTGATACATTACCGATTGATTCCGTTACGCGGATGACACAAAATATGTCAGTTTAGCACCGGCCGCGCTCCCTTTCCCGGTTTGTTCGGCGACGCGGGGGGAAGGGAGACCGAGCGCACATTAGCAGGGAAGGTGACAGCGGAGGCTTCACCCATCTCTCTGTCTCTCCGGATCCAGATGTTGGCTTTAAGGTTTCTCCAAACCTAGAAATCTGTTGAAGATATTACTTCTTTGCGAATCTCTCTTTGTTCTTGAATCGGGTCCAGCGCGGAAGTAGCTGTCGTCAGTAGTAGTTGTGATTCGTGGGAGTTTGAGTTTGATTGGAAGTCCAGTAGTAGTGAGATTCTGCGCCGATCGAGATG

MapGlcT4-1 (XP_009401350.1) (Ma05_p26070.1)

TCGCCTTCGTGTGGGGTTGAAGGGGATTTGGATGGGATGAGGAAGAAGGAAAGAGGAGGAAAAGAAGGCGGTGGTCGGAGGAGGAGAGAGAGTGGCCGACGACATGTGGCTGCGTGTGGCCGCTGCGCCTCCGCAGTTTTCCTTTTCGGTTGTCGAACCGCAATGACAACAGAAATGACAGCAAATTATATAATAATATTATTATTTTATTTATTTTTAATATTAATAATAAAACAATAATAATAATAATATTATTATTATTATCGAATTCACTTCCCAAACATATCCGATGTTATTGTATTAAATAATTAAAAATATTTTTAAAGATATTTTTAATGTGCATTCGGATAATGTGCATATTTATATCTTAAATATGAAGTGATGTAAATACTATTGGTTAAAATATATTTAATTTTTTAATACATATTATATAATAAAATTTATTATTATAATATTTTCAAATATTTATTTTTAGAAAATATATATATTTTTAAAATTAATAAGTAAATAAAAATAAACATACTCTTAAAAAAAAATTCATATTTAACTGATAAAATTATTTGATTATAAATAGCATTTCCATGGGATAGAATCTTATCTTTATCAATTATTTTAAAAAGAAATAATAATAATAATAATTAATAAATAATATTGAGGAAACTATCTGTTTATTCTTGAATCTAACTACTTAAGTATTTGAATTAGATTAATCTAATTTCAATCCGATTTTATCTCCACCGAAATAGTGTATTGTTGAAAAAAGATTAAGGAAAATTATAATAAAACATACTTAAATTTTATAGTTAATGTTGATTGACTAGTCATTTAATTGTTATTTGACACTCTAATTGAGGTTTATATCTTTCCAAAATTTTATTATGATATAAAAGTACATGCATTTAACTCAAAAAAATTAATATTAGTGGTTAATATCTTTTTATCACCCAAACTAACTAATATTCTAAGAAAAAATTATCAAATGAAATATAAATTTTTTTACTTTTACTATAGAAATGATTAATTTATGATAACATCAATATTTACAGGGGAATATAATATTCACATGAAATTTATAAGGATAAATACACAATTTTGAAATAGATGGAAATATACGTTAAAACCTCTAAATCATTAATAGAAACATTAATATATAACATATATTGGAGATTAATCGATCTGTCACAGATATAGTAATACCAAAATCTCGTGCGACCGTCCTTATCATGTGACACGTATGCAGTATTGGACCTTTTCCCCTGCACTGTGGAATCCAAACCCGCCACTTGCGCTCTCTCTCTCTCTCTGTCTCTCTTCCGTCGATGAGAGCGGATCGTTCCAAGCTTCTGCTACTCTGTTTTGGAGTCTCTCTCTCTCTCTCTCTCTCTCTCGCTCCTGTCGACGCCCACAAGGGGATTCTTTGCGCTTCGGACTAGCTTGATCGGTAACGGCTCTCAAAGATCGATCTTTTCCTCTCTTTCCTGACCCATTTGAATCGATTGTTTCGATTATTGAGTGCCAATCTTTTGCTGTAAGGCAAACGTCGTCTTTTTGGGTGATTTCTCTATGTTGCTGTTGTTTTATTGGTCTGTCGAGATCAGAATTGTCCCCATCAATGCAGGCGCTTTCAATCGTTCCTATTTTTTTTTACTATGTCTATTTATGTGACAGGAGGGGATTAGAAGGGAAAGATG

MapGlcT4-2 (XP_009380955.1) (Ma10_p25440.1)

GGATACAGGAGTCACCATACTAGACAGCAGGTTATCAACGGATGGCATCGAGAACTCCGGCTGATGAGATAAGCAGTTCTGTTGAGGATAGCACATCAACTCCAAGCTTGAATTCTCCATGGCAGGGCCTTCTAGCTCTGGAAACTTGCGGGTGAGCTCCAAGCCGGAGTCCTCAAGAAACTCTTCCATGACTACAGGAAAGAGAAAACAAGCGCCTAAAAGAAAGTGTTTGGATGAGAAAGGAGGCCTTTAGAGTAGGCCTGAACAAGTTCTTCCACCACTATATGAATGCGATGTGTAGAGCAGCTCTCGAGCAGATCAACTTGGCAAATCGAGTTTGTGTAGTCGGCTTGTGGGTGCTCACGGAACACATCTACAGCTCTTGGACATATATGTGCTCCAAAATCTGACCATTAGATTCAAGCAGTAAATGAGCATTAATTTGTTCTTTTTGCTCTTGAAGGAAAGTAGAAAACAGATTGCTTTTACAGTATTTTGGTTTGTGTCAGCTCTTCCTTGAGACTCATACTCTGCTTCAGTGTTCGTCTCCCAATCCAAAGAAAATACTGCAGAAGGGCATCTAACATAGTTCGTACAAGTTGACGATGCTGTGTAGATGGTTAGAACTCGTCAATCACAGGGCAGGAGGTTCACCTGTTTTAATCAGTGATCACTAAAGAGAGCCAATGAAGACAAAACCAAACCCCAAAACGAGATCTATGTAAACTTGTTAGAAAGATCGAGTATCTACAACGAGGACACTGATCGATGAAGATATCGCCGATTGTTACTGAAAGAACTTAGCTTGTTTTGGTGTATGAGGTAAAGCGGTTCGAGTCTCTTTTCATGCACACCTCTTGGATCTTCCTGTTGTACGCGGTTAGATTCGACTAGGTCGTCGCACGCGCAGCACACTTGCCTGAGACGGCGATCTGATTTCTTGACCTTTCGATTCATGACCAAAAGCAAGTTTGTCATGGAATTTAGCATGATCTTAGGGGTGTGTGTTCCGATGACTATAACAAATCTATACACCATTTCATCTTCTGCGATCCACGCATCATAGATCTTCTACCGACATGAGCAAACAATGCAAAGGAGCGTTCGTTCATCTCGGATCCTCCCGCCGTACGCCGCCGCAGCGGTTGGAAGGCGCGTACGGCCGTACACGTACACAGAATCCAACACCTCCCACCGACCTAAATCCAGACTCCCTCTTCCTCCTCCTTTCTCTCACTGATCTTTCCACCACAGGACGGACGCCCGACATGGCGTCATTCTTGCAACTCCCCGCTGCCTTCCCTACTTCTCGGTAACAAAATAATCTCCCTGTCAAGATTGCTTCTTCCGTCAAAATGGCGGCGATCGCGAAGCTTCTGTTGCTTCATTCTCTTTCTCTCGCTTCTCGATCTCAGCAAACGGAGGTGTGGAGGATACGTCAAAAGTATTGTCTTTCCACTTCGGATTAGATCGATCGGTGGAAAATTCTGCAAGATCGCCCCCTTTTTCTATCTTTGCAGCATTTACATCCGTCTTCTTGCCAAATTTCTCTAAAAGTCAACTTTCCATTAACAAGAATAATAATAATAAATGGAATGGCCTGTCTTCGTTGCTATTTCGTTCCTCGAGATCCGAATTGTCCCTGTCACCAAGGCGGTTTCGATCTGGTTTCTTTCTTCTGTATTTATGACAGGAGGGGATTCGTAGCGAAAGATGGTGCACAACGGTGTGGTCGCTGCCTTTGAGTTTCCAGGTCGCGGGACAGGGTTTAGGCTGCGCTTGGCTTGGTCAAGAAGAGGAGCGCAAGATCGAACGGGTTGTCGATG
